# Long Term Follow-Up after a Randomized Integrated Educational and Psychosocial Intervention in Patient-Partner Dyads Affected by Heart Failure

**DOI:** 10.1371/journal.pone.0138058

**Published:** 2015-09-25

**Authors:** Maria Liljeroos, Susanna Ågren, Tiny Jaarsma, Kristofer Årestedt, Anna Strömberg

**Affiliations:** 1 Department of Medicine and Health Sciences, Linköping University, Linköping, Sweden; 2 Centre for Clinical Research Sörmland, Uppsala University, Uppsala, Sweden; 3 Department of Cardiothoracic Surgery, County Council of Östergötland, Linköping, Sweden; 4 Department of Social and Welfare Studies, Linköping University, Linköping, Sweden; 5 School of Health and Caring Sciences, Faculty of Health, Social Work and Behavioral Sciences, Linnaeus University, Kalmar, Sweden; 6 Palliative Research Centre, Ersta Sköndal University Collage and Ersta Hospital, Stockholm, Sweden; 7 Department of Cardiology, County Council of Östergötland, Linköping, Sweden; University of Pennsylvania, UNITED STATES

## Abstract

**Background:**

To date, contemporary heart failure care remains patient-focused, but awareness of the partners’ and families’ situation is increasing. Randomized studies have mainly evaluated the short-term effects of dyadic interventions. Therefore, the aim of this study was to determine the 24-month effects of an intervention with psych-educational support in dyads of heart failure patients and their partners.

**Methods:**

This study used a randomized study design and 155 patient-partner dyads were enrolled. The intervention included a nurse-led program of three sessions addressing psychoeducational support.

**Results:**

The intervention did not have any effect on health, depressive symptoms or perceived control among the patient-partner dyads after 24 months. Furthermore, time to first event did not differ significantly between the intervention group and the control patients.

**Conclusion:**

This study may be regarded as a first step in trying to understand dyads’ need for supportive care. Individualized and more targeted interventions seem necessary to achieve a higher impact on dyad outcomes.

**Trial Registration:**

ClinicalTrials.gov NCT02398799

## Introduction

Heart failure (HF) is a serious condition with poor prognosis. It is the leading cause of hospitalization, and readmissions for worsening HF remain high [1]. Despite the fact that most patients receive education in managing their HF, deterioration is often the result of patients’ t non-adherence to treatment [2–4]. Numerous barriers, both physical and psychosocial exist that affects patients non-adherence, for example cognitive deficits, depression and other co-morbidities, which make it difficult for many patients to learn to perform adequate self-care [5–7].

The care for patients with HF mostly takes place at home and partners provide the main assistance. Partners form an important resource in supporting patients’ self-care, such as medication adherence, symptom monitoring, diet changes and exercise [8–11]. Partners are often the first to notice new symptoms, and many health problems are handled by patients and partners without consulting healthcare professionals [12]. At the same time, it should be acknowledged that HF could also affect partners negatively [13]. Taking care of an ill or disabled individual imposes a well-documented burden on the caregiver, both in health effects and quality of life [14,15]. Emotional reactions, such as burden and stress, decrease when partners experience that they are in control of their heart disease [16–18]. Therefore partners should also be included in HF management programs [19,20].

Despite the fact that HF has a number of negative consequences for patients and partners, previous research has mainly focused on improving patients’ outcomes. However,awareness of the partners’ and families’ situation is increasing.

Martire et al [21] conducted a meta-analysis of psychosocial interventions for chronic illness, targeting patient-partner dyads as well as patients and other family members. The interventions focused both on patient outcomes (depression, anxiety, relationship satisfaction, disability, and mortality), and family member outcomes (depression, anxiety, relationship satisfaction and caregiver burden). The meta-analysis found that the interventions had positive effects on depression and, in some cases, on mortality in patients. Among partners and family members, there were positive effects such as decreased caregiver burden, depression, and anxiety when interventions addressed relationships issues. These interventions resulted in less depression and burden among the closest family members.

A systematic review by Reid and colleagues [22] concluded that psychological interventions among patients with coronary heart disease and their partners decreased blood pressure and improved quality of life in patients, and also decreased symptoms of anxiety in partners. Knowledge of and satisfaction with care were improved in both patients and partners.

However, patient-partner dyads, or patients and other family members, have only been included in a few RCT studies on the HF population, and most studies have only evaluated short-term results [20,23–25].

A Shared Care Dyadic Intervention focusing on communication, decision-making and reciprocity was recently tested in patients with HF and their partners [23]. Eleven dyads met in seven sessions over a period of twelve weeks, with the aim of helping them to exchange support and assistance. The result at the end of the intervention showed hardly any improvements in communication and decision-making in the patient group, but the partners seemed to benefit more.

A family-oriented, educational program showed no significant differences between the intervention group and the control group with regard to anxiety, depression or quality of life. The intervention group, which included only family members of patients with HF, but no patients, met five times over a period of six months. The authors concluded that improved disease-related knowledge might need to be combined with other target variables to induce the desired effects [24].

Dunbar and co-workers performed a three-arm randomised trial where they tested a family-patient education intervention, a family partnership communication intervention, and usual care. Both shared education and family partnership communication reduced dietary sodium intake over four to eight months, compared to usual care. However, the patients’ medication adherence, knowledge about HF and perceived autonomy support did not improve and family criticism did not decrease [25].

The lack of research on heart failure dyads inspired us to develop and test an intervention that combined education and psychosocial support in dyads of patients with HF and their partners. Short-term results showed significant differences in the patients’ perceived control over their HF but no effect was detected among the partners. As for the dyads’ health-related quality of life and depressive symptoms, there was no effect over time until 12 months [26].

The aim of the current study was to determine the 24-month dyadic effects of a supportive educational program for patients with HF and their partners. We hypothesized that the intervention could reduce morbidity among patients with HF, as well as improve health, depressive symptoms, and perceived control among HF patient-partner dyads in the long-term.

## Method

### Design

A randomized controlled design with a follow-up assessment after 24 months was used to evaluate the effects of a twelve-week educational and psychosocial intervention, delivered in three sessions to patient-partner dyads affected by HF. The study was registered at ClinicalTrials.gov Identifier: NCT02398799 after patient recruitment began (S1 Table). The reason for this is that when recruitment began, it was unusual to register this type of intervention studies. The intervention design and results at three and twelve months follow-up data, reported for patients and partners separately, have been published elsewhere [26]. This paper focuses on the long-term effects and will analyze the data on a dyad level with patients and partners combined and treated as equals.

### Sample and settings

The sample included dyads consisting of a patient and a partner who acted as the informal caregiver. The inclusion criteria were: being a dyad consisting of a patient diagnosed with verified HF according to the European Society of Cardiology guidelines [27], in NYHA class II-IV, recently discharged from hospital (i.e. two to three weeks) following acute exacerbation of HF, and cohabiting with a partner in a marriage-like relationship. Exclusion criteria were: diagnosed dementia or other severe psychiatric illnesses, drug abuse, difficulties for one of the dyad members to understand or read the Swedish language, planned cardiac surgery, or participation in other studies.

A power analysis was conducted to justify the sample size. Lack of evidence for clinically relevant improvements in the outcome variable scores made it difficult to estimate a relevant difference score between the intervention and control group. Therefore, we used a pre-defined medium effect size for regression models. With an expected medium effect size (*f* ^2^ = 0.10), a statistical power of 1- *β* = 0.90, and a 5% significant level, the estimated sample size was determined to 108 participants, 54 in each group. As the statistical models needed to be adjusted for the fact that patients and partners were nested within dyads, the sample size was doubled. Thus, a sample size of 216 individuals (108 dyads) was expected to be sufficient. For the inclusion at baseline, an additional 94 individuals (i.e. 47 dyads) were added as the dropout frequency was expected to be high during the 24-month follow-up period.

The participants were recruited between January 2005 and December 2008 at one university hospital and one county hospital in the southeast of Sweden. All patients diagnosed with HF admitted to the hospitals for deterioration of HF were screened. The dyads were initially informed about the study verbally by telephone, or during a visit to the heart failure clinic two to three weeks after hospital discharge. Patient-partner dyads that were interested in taking part in the study and provided written informed consent were given additional information and questionnaire packets to complete at home. Dyads choosing to participate returned the questionnaires by mail and were then randomized to either the control or experimental group. The randomization codes were generated using a random number table.

### Procedures

#### Control conditions

The dyads in the control group received care as usual, both in the hospital and the outpatient clinic. Care as usual included optimized treatment according to international guidelines, and verbal and written patient education. Standard care focused on the patient’s needs, and although partners were able to join in, they were not systematically invited to participate in the care [27–29].

#### Intervention conditions

The dyads in the intervention group received care as usual. In addition, they participated in an educational and psychosocial intervention, which included psychosocial support to maintain and strengthen the dyads’ physical and mental functions and perceived control.

The theoretical framework for the study was based on a conceptual health promotion model developed by Stuifbergen et al [30,31]. The model focuses on enhancing self-efficacy and has successfully been used as an educational program with supportive telephone follow-up.

The authors describe that barriers and resources can be enhanced by education and support, and help individuals to participate in health-promoting behaviors, such as self-care.

The intervention was delivered in three modules through nurse-led face-to-face counseling, a computer-based program and written materials. The sessions took place two, six and twelve weeks after discharge from hospital. Each of the three modules contained cognitive, supportive and behavioral components and outcomes (see Table 1). All sessions included education on heart failure and development of problem-solving skills to assist the dyads in recognizing and modifying factors that contribute to psychological and emotional distress. The intervention focused on changing thoughts and behaviors, and implementing strategies for self-care behaviors.

**Table 1:** Description of the modules in the intervention.

	Module 1	Module 2	Module 3
**Cognitive Component**	The circulatory system, definition of HF, medications and symptom management	Lifestyle modifications; diet, smoking cessation, alcohol, immunization, regular exercise	Directing the care, relationship and prognosis
**Cognitive Outcomes**	Increased knowledge of the chronic HF syndrome and treatment	Increased knowledge of the rationale for lifestyle changes	Increased knowledge of self-care and outcomes
**Support Component**	Introduce psychosocial support concept	Assess patient’s need of support, Modify caregiver behavior	Assess partner’s need of support and partner’s caregiver burden
**Support Outcomes**	Improved mental and physical functions	Strengthen self-care behaviour	Improved mutual support Decreased caregiver burden Improved control
**Behavioural Component**	Intentions, abilities and self-efficacy regarding self-care	Barriers to lifestyle modifications	Strategies to improve or maintain self-care behaviour
**Behavioural Outcomes**	Daily weighing, Monitoring of symptoms, Flexible diuretic intake, Adherence	Salt and fluid restriction, Influenza and Pneumococcal Immunisations Regular Exercise	Identifying life priorities and planning for the future

Content of each of the three modules utilised in the intervention.

Briefly, the first visit aimed at increasing the dyads’ knowledge of the disease and treatment, improving mental and physical functions, and introducing self-care behaviors such as daily weight monitoring, adherence to medication and a flexible diuretic intake.

The second visit aimed at increasing knowledge of the rationale for lifestyle changes, assessing the patient’s need for support, modifying and strengthening caregiver behavior, as well as identifying barriers for lifestyle changes.

The third visit focused on increasing knowledge of heart failure care and outcomes. It was a reinforcement of the intervention, and included an assessment of outcomes on support, behavior and repeated computer-based education. The visit also assessed the partner’s need for support and perceived caregiver burden, in order to find strategies to improve control and self-care behavior, and plan for the future.

The sessions were conducted in the dyads’ homes or in the heart failure clinic, depending on the dyad’s preference. Each session lasted approximately 60 minutes.

All nurses were experienced HF nurses who had received special training on how to perform the intervention before the study. The nurses received three days of theoretical training followed by individual and practical training. To ensure accuracy of the intervention, the study team regularly assessed the nurses’ competence to deliver the intervention through observations and consultations.

### Measures and data collection

Separate questionnaire packets to be completed at home at baseline and after 24 months were sent to the dyads by mail. Patients and partners filled in separate questionnaires and were instructed not to discuss the questionnaires with each other. All questionnaires were filled in by both the patient and the partner. If the patient or partner in a dyad died, or could not fill in the questionnaires at 24 months, the other person did not fill in the questionnaires. Demographic and health history data regarding education, smoking habits, alcohol consumption, physical activity, psychosocial support and co-morbidity were collected using a self-administrated questionnaire. Data on time to first event, readmission or mortality, were collected from patients’ records. Due to the nature of the intervention, the study could not double blinded. However, both data collectors, and researchers entering the data were blinded to group assignment.

The 36-Item Short-Form Health Survey (SF-36) was used to assess health in eight dimensions, and for a physical and mental component summary. The dimensions include physical functioning (PF), role limitations due to physical health problems (RP), bodily pain (BP), general health (GH), vitality (energy/fatigue) (VT), social functioning (SF), role limitations due to emotional problems (RE), and mental health (psychological distress and psychological well-being) (MH). For each of the eight dimensions, scores were coded, summed and transformed to a scale from 0 (worst possible health) to 100 (best possible health). The SF-36 has been frequently used and has been found to have good reliability and validity [32,33].

The Beck Depression Inventory II (BDI-II) is a self-rated instrument with 21 items assessing different symptoms of depression. Each answer was scored on a scale from 0 to 3. Higher scores indicated more severe depressive symptoms. The constructors’ recommended cut-off scores are: 0–13 (no depression), 14–19 (mild depression), 20–28 (moderate depression), and 29–63 (severe depression). There is a validated Swedish version of the instrument. In this study, the internal consistency reliability at baseline was α = 0.92 in the patients and α = 0.90 in the partners [34].

The Control Attitude Scale (CAS) is a tool consisting of four items designed to measure an individual’s perceptions of control over their cardiovascular disease. The CAS was also used in the partner group. Response statements were scored on a scale from 1 (none) to 7 (very much). The total score range was 4 to 28, with higher scores reflecting higher levels of perceived control. Reliability testing in different language versions has revealed satisfactory internal consistency [35–37]. In our study, we found Cronbach’s alpha values to be >0.80 for both the patient and the partner version at baseline.

### Ethical considerations

The principles outlined in the Declaration of Helsinki were followed throughout the study, which was approved by the Regional Ethical Review Board in Linköping, Sweden (Study code 03-568/ M178-04). All dyads received verbal and written information about the study and those who chose to participate gave written informed consent before entering in the study.

### Data analysis

Missing data in the SF-36 was imputed by the means of the subscale if only one item in the subscale was missing. Missing data in other instruments were not replaced since this was not recommended by the constructors.

Depending on the level and distribution of data, group comparisons were tested by chi-square statistics, Fischer’s exact test, Mann-Whitney *U-* test, or Student’s *t*-test.

The Kaplan-Meier survival analysis was used to examine the distribution of time between the first event, number of days to readmission, or death among the patients. The log-rank test was used for comparing the Kaplan-Meier curves between patients in the control and intervention group [38].

Linear regression analyses were conducted to determine the effect of the intervention on health, symptoms of depression and perceived control. For each outcome variable, the difference in scores between baseline and the 24-month follow-up were used as dependent variables, whereas group affiliation (intervention or control) was used as an independent variable. In these analyses, both patients and partners were included together at the same time.

Based on the hierarchical structure with patients and partners nested in dyads, regression analyses with robust variance estimates were used [39]. The same linear regression analyses were also conducted on patients and partners separately, to further investigate intervention effects within these groups.

The results was analyzed using intention-to-treat analysis including all randomized dyads. Since all dyads attended all three modules of the intervention ‘per protocol’ analysis were not needed.

The level of statistical significance was set to *p* <0.05. The statistical analyses were conducted using SPSS 18 for Windows (SPSS Inc, Chicago, IL, USA), and Stata 12.1 for Mac (Stata Corporation, College Station, TX, USA).

## Results

### Characteristics of the patient-partner dyads

**Fig 1:**
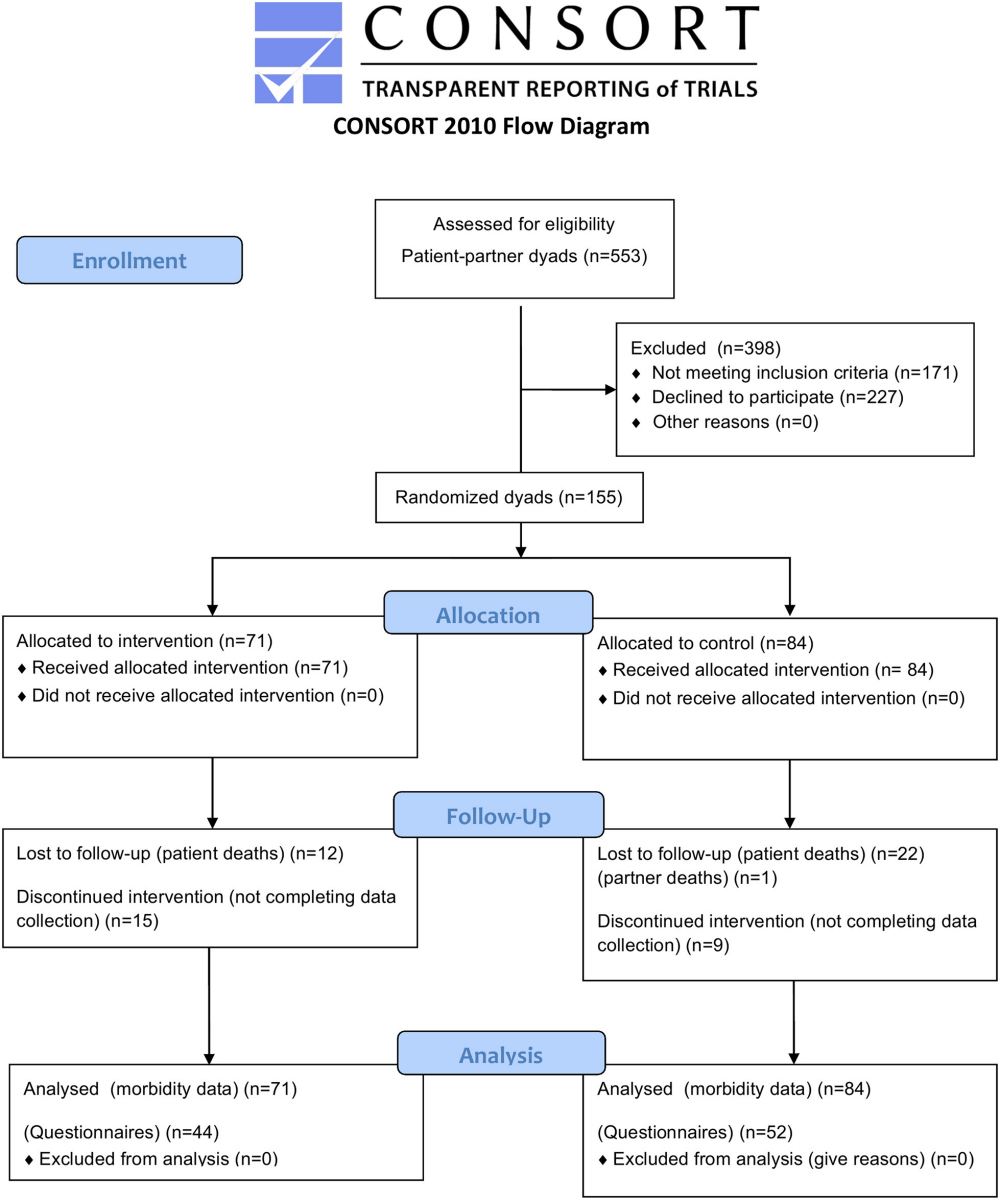
Flowchart for the participating dyad from enrolment until 24 months.

The final sample consisted of 155 dyads of patients with HF and their partners. There were 142 patients and partners in the intervention group and 168 in the control group (Fig 1).

Clinical and demographic characteristics of the dyads are described in Table 2. The majority of the patients were men and treated with HF medications according to guidelines [1]. Most patients were in NYHA functional class III.

**Table 2:** Clinical and demographic characteristics of the patient-partner dyads at baseline.

	Control		Intervention	
Characteristics	Patient (n = 84)	Partner (n = 84)	Patient (n = 71)	Partner (n = 71)
Age, mean±SD	72.9±10.1	69.5±10.5	69.4±13.6	67.1±12.1
Female, n (%)	16 (19,1)	68 (80,9)	22 (30,9)	49 (69,1)
**Medical history, n (%)**				
Myocardial infarction	38 (43)	13 (15)	23 (33)	8 (11)
Hypertension	26 (31)	25 (30)	27 (38)	25 (35)
Diabetes	10 (12)	4 (5)	8 (11)	7 (10)
Stroke	8 (10)	4 (5)	9 (13)	3 (4)
Lung disease ^a^	7 (8)	10 (12)	3 (42)	1 (1)
Pacemaker	9 (11)	1 (1)	11 (15)	1 (1)
Cardiac resynchronization therapy	15 (18)	1 (1)	8 (11)	0 (0)
Implantable cardioverter defibrillator	2 (2)	0 (0)	3 (4)	0 (0)
Percutaneous coronary intervention	15 (18)	3 (4)	10 (14)	3 (4)
Coronary artery bypass surgery	17 (20)	3 (4)	12 (17)	2 (3)
**NYHA class, n (%)**				
II	25 (30)		24 (35)	
III	43 (51)		40 (55)	
IV	16 (19)		7 (10)	
**Medication, n (%)**				
ACEI/ARB	76 (90)		65 (92)	
Beta-blockers	74 (88)		62 (87)	
Diuretics	63 (75)		56 (79)	
**Education, n (%)**				
Elementary school	56 (65)	48 (58)	40 (59)	41 (59)
High school	21 (26)	22 (28)	22 (32)	26 (37)
University	7 (9)	14 (14)	9 (9)	4 (4)
Years at school, mean±SD	9.8±6.1	9.9±3.5	9.4±4.7	9.7±3.2
**Employment, n (%)**				
Full time	10 (11)	18 (20)	10 (14)	22 (33)
Disability pension/sick leave	10 (11)	4 (5)	13 (17)	2 (3)
Homemaker	3 (2)	0 (0)	1 (1)	2 (3)
Pension	61 (76)	62 (75)	47 (68)	45 (61)
**Lifestyle, n (%)**				
Smoking/ Ex-smoking	47 (60)	39 (49)	36 (54)	30 (42)
Alcohol Never drink alcohol	20 (26)	18 (23)	16 (24)	15 (23)
≤7 glass/week	54 (71)	57 (74)	45 (67)	46 (69)
> 7 glass/week	3 (3)	2 (3)	6 (9)	5 (8)
Unknown	7	7	4	5
Exercise < 30 min/week	27 (36)	6 (8)	13 (20)	4 (6)
30 min-3 hours/week	25 (31)	28 (36)	36 (42)	29 (43)
> 3 hours/week	26 (33)	43 (56)	19 (28)	35 (51)
Unknown	6	7	3	3
BMI, mean±SD	26.8±4.1	26.8±4.1	26.6±4.5	26.6±4.9

^a^ Lung disease was significantly (*p* <0.05) more common in the partner control group compared to the partner intervention group.

There were no differences between the patients or partners in the control and intervention group at baseline, although lung disease was significantly more common in the partner control group (*p* <0.05).

As shown in Fig 1, a total of 59 dyads did not complete the 24-month assessment. During the follow-up period, the all-cause mortality rate for patients was 17% (n = 12) in the intervention group and 26% (n = 22) in the control group. One partner in the control group died during the 24-month follow-up. A total of 15 dyads in the intervention group and nine in the control group were too frail to complete the questionnaires at 24 months. There were no significant differences between the responders and non-responders regarding age, gender, education and employment, neither in patients or partners. The 24-month assessment was based on 44 dyads (patients and partners n = 88) in the intervention group and 52 dyads (patients and partners n = 104) in the control group.

### Patient events

The Kaplan-Meier curves did not differ between the groups (*χ* ^2^(1) = 1.10, *p* = 0.293) with regard to time to first readmission/death. There was no significant difference between the groups in number of days to the first readmission, or the number of short hospital readmissions. Events (readmission/death) within 24 months occurred in 72% of the patients (n = 51) in the intervention group and in 68% (n = 58) in the control group. In the control group, 77% of readmissions were due to heart failure or heart disease. In the intervention group, this figure was 87% (Table 3).

**Table 3:** Readmissions and reason for readmissions during the 24- month’s follow-up period.

	Control patient, n = 84	Intervention patient, n = 71	p-value
Readmitted 24 months, n (%)	58 (68)	51 (72)	0.72
Days to first readmission, mean (SD)	403.5±282.5	345.6±282.7	0.09
Number of readmissions, mean (SD)	1.6±1.6	1.8±1.9	0.70
Number of days in hospital, mean (SD)	4.0±14.0	4.0±15.0	0.50
Hospitalization ≤2 days, n (%)	19 (37.3)	21 (44.7)	0.54
Hospitalization >2 days, n (%)	32 (62.7)	26 (55.3)	0.41
**Reason for readmission**			
Heart failure, n (%)	33 (34)	31 (38)	0.64
Heart disease, n (%)	41 (43)	41 (49,3)	0.52
Other reason, n (%)	26 (27)	28 (34)	0.29

Student’s t- test was used for continuous variables, presented as mean ± standard deviation (SD). Categorical variables are given as percent (%) and are compared using Chi-square test.

### Patient and partner reported outcomes

The intervention did not have any effect on health, depressive symptoms or perceived control using dyadic analysis among the patient-partner dyads after 24 months (Table 4). To further explore if the intervention had specific effects on patients or partners, regression analyses were conducted on each group separately. The analyses did not show any significant differences between the patients in the intervention and control group.

**Table 4:** Multiple linear regression analyses (robust variance estimates) to detect intervention effects regarding health, symptoms of depression and perceived control for both patients and partners.

Outcome variables	Group ^a^	Mean diff (SD) ^b^	β (SE) ^c^	95% CI for β	p-value
PCS (n = 183)	Intervention group	-2.67 (0.93)	-1.08 (1.31)	-3.68 / 1.53	0.415
	Control group	-1.60 (0.96)			
MCS (n = 183)	Intervention group	3.49 (1.10)	0.94 (1.79)	-2.61 / 4.49	0.601
	Control group	2.56 (1.20)			
PF (n = 189)	Intervention group	-4.28 (2.26)	-2.80 (2.83)	-8.41 / 2.81	0.325
	Control group	-1.48 (1.88)			
RP (n = 187)	Intervention group	-3.50 (4.57)	-1.82 (6.42)	-14.56 / 10.92	0.777
	Control group	-1.68 (4.12)			
BP (n = 190)	Intervention group	-3.33 (2.91)	-2.56 (4.67)	-11.83 / 6.72	0.586
	Control group	-0.77 (3.04)			
GH (n = 190)	Intervention group	-0.18 (2.07)	2.40 (3.01)	-3.58 / 8.37	0.428
	Control group	-2.58 (1.85)			
VT (n = 189)	Intervention group	5.23 (2.18)	2.34 (3.25)	-4.11 / 8.78	0.473
	Control group	2.89 (2.19)			
SF (n = 190)	Intervention group	1.40 (2.36)	-3.17 (3.53)	-10.19 / 3.84	0.371
	Control group	4.58 (2.48)			
RE (n = 186)	Intervention group	7.66 (4.32)	2.61 (6.25)	-9.80 / 15.02	0.677
	Control group	5.05 (4.15)			
MH (n = 189)	Intervention group	3.30 (1.89)	0.43 (3.01)	-5.56 / 6.41	0.888
	Control group	2.87 (1.89)			
BDI (n = 133)	Intervention group	0.66 (0.68)	-0.06 (1.34)	-2.74 / 2.63	0.967
	Control group	0.71 (0.98)			
CAS (n = 183)	Intervention group	2.33 (0.52)	0.65 (0.76)	-0.85 / 2.15	0.395
	Control group	1.69 (0.48)			

^a^ Control group as reference category.

^b^ Mean difference between the baseline and 24-month follow-up assessment.

^c^ Robust standard errors (robust variance estimates).

PCS = SF-36 physical component scale, MCS = SF-36 mental component scale, PF = physical functioning, RP = role limitations due to physical health problems, BP = bodily pain, GH = general health, VT = vitality, SF = social functioning, RE = role limitations due to emotional problems and MH = mental health, BDI = Beck Depression Inventory, CAS = Control Attitude Scale.

As for the partners, both the intervention and control group reported decreased physical health between the baseline assessment and the 24-month follow-up. However, those in the intervention group had a significantly greater decrease in both PCS (*B* = -4.13, *t*(90) = -2.43, *p*<0.05), and physical functioning (*B* = -6.76, *t*(93) = -2.21, *p*<0.05). No other differences were identified between the partners in the intervention and control group.

## Discussion

This is, to our knowledge, the first study which report on patients’ event-free survival and the long-term effects of an integrated educational and psychosocial intervention on a dyad level in patients and partners affected by HF. Dyadic intervention is still a young research field and this intervention may be seen as a first step in trying to understand dyads’ need for supportive care.

What is unique about this study is that patients and partners participated as equals and data were analyzed on a dyad level. As we included couples, we anticipated violations against the assumption of independency. It is known that dyads influence each other and therefore we intervened and analyzed outcomes of the intervention for the dyad together. Doing a dyadic analysis instead of separate analysis for the patient and partner separate was one way of controlling for the independence.

HF guidelines [1] advise that partners be involved in the care, but it is currently not known how these interventions should be designed to achieve the best outcomes. Despite a well-structured and theory-based intervention, the effect over the 24-month follow-up period was minor among the dyads. Event-free survival, mortality, or hospitalization did not differ significantly between the groups. This might be due to the fact that patients in both groups received evidence-based treatment and structured follow-up at a nurse-led HF clinic, which is known to decrease morbidity and mortality [27,40]. Peters-Klimm et al [41] found that a one-year case-management program for patients did not improve health outcomes in a medically well-treated HF population, but that self-care behavior improved. In our study, some partners in the control group may have accompanied the patient to their visit to a nurse-led HF clinic. This might have influenced the contrast between the groups, where mental health and perceived control improved in both groups.

Our patients experienced low perceived control at baseline, which improved to a moderate level during the follow-up period in both groups. The level of depressive symptoms was low both at baseline and at 24 months. Due to these findings, it may be difficult to expect dyads to improve their scores. Patients with higher levels of perceived control experienced less depression and less anxiety compared to patients who experienced lower levels of perceived control [35].

Regarding the lack of effectiveness of the intervention there might be several hypothetical explanations, such as the insufficient content of the intervention which was psycho-educational and that may not have been sufficient in terms of fulfilling all the needs of the dyads. To improve outcomes, individualized and more targeted interventions, which address both practical and mental components, are probably needed [42]. Practical education and information may facilitate patients and partners in experiencing increased control and sharing care, as has been reported in cancer care. Active partner participation can enhance self-care behaviors and increase partners’ perceived security. When symptoms occur, the patient often consults a family member first, and they are the ones who support medical treatment adherence. If partners do not have knowledge, or understand how to support lifestyle change, adhering to self-management activities may be difficult for the patient [20].

An insufficient ‘intensity of the intervention (too short or too few sessions) may also explain the lack of effects of the intervention. The length and dose of the intervention influence outcomes. Earlier studies have found that short interventions might not be enough to change outcomes [43]. In the current study, patients scored higher levels of perceived control at the 3-month assessment, but the same result was not found after 12 months [26]. Perhaps, a 12-week program is not enough to achieve a long-term effect on perceived control. Studies that have used longer intervention periods have been more effective [44]. An intervention consisting of six weekly two-hour sessions of dyad-oriented group education and support intervention in osteoarthritis patients and their partners showed that after six months patients participating in the couple-oriented intervention reported greater increased spouse support than those participating in the patient-oriented intervention. Partners also perceived a higher level of control [45].

Birnie et al. [46] showed that an extensive eight-week and eight-session mindfulness-based stress reduction program (MBSR) improved psychological functioning in dyads of cancer patients and their partners. The program reduced mood disturbances and stress, and the result indicated that when one member of the dyad is distressed or perceives a loss of control, the other is likely to do so as well.

Before designing new interventions, there is a need to learn more about dyads’ need for supportive care and maybe different methods should be combined to achieve better results. Both patients and partners describe a need for continuous guidance and easy access to healthcare providers during the whole illness trajectory, not just for a limited time after diagnosis or hospitalization [47].

The current study included an educational and psychosocial intervention and conflicting results have been reported regarding the benefit of psychosocial interventions for families.

Cheng and colleagues [48] recently systematically evaluated eighteen studies of stroke family caregivers and stroke survivors and concluded that evidence concerning the effects of psychosocial interventions was limited and more research is needed.

On the other hand, a psycho-educational program involving relaxation techniques for family caregivers of people with Alzheimer's disease found that the program was beneficial for caregivers' burden and mental health status [49].

In the current study only the patient and the care-giving partner were included, but also including other family members can be one way to achieve better outcomes. A broader approach where patients choose their participating family members may be beneficial. If patients select the family members he or she wants to be included, they may have better effects from interventions, as people living together are not always are the ones supporting each other.

Our study has some limitations. Despite the fact that we included a large number of additional dyads at baseline, the dropout frequency was high due to death and frailty. Therefore, none of the regression analyses included the required sample size determined in the power analysis. However, this probably did not affect our conclusions as the mean differences between the intervention and control group were small for all outcome variables. The reason for the large share of dropouts and frequency of missing data are probably multifactorial.

Many of the eligible patients or partners found during the screening were too fatigued or marked by illness or multi-morbidity to participate in this type of intervention that requires an active commitment from the dyads. This suggests a potential for selection bias in the study sample, as only 28% of those eligible were randomized. Dyads declining participation have been found in other studies as well. The most common reason for refusal was living far away from the hospital, partners not wanting to participate, patients feeling too ill or not wanting to put another burden on their partner [50].

In many other studies, the prevalence of depressive symptoms has been higher for both patients and caregivers. However, having included more patients with depressive symptoms would probably not have affected the results since a review article by Woltz et al. [51] found strong evidence that disease management programs do not improve outcomes in depressed patients with HF.

The large number of items in the questionnaire package could be tiring for elderly people to complete, and maybe the intervention was not fully aligned with the evaluated outcome. In this type of complex interventions it is always a concern not to have chosen sensitive outcomes that mirror the content of the intervention [52]. We used a variation of outcomes as recommended for complex interventions. However, there are a number of other related variables, and other instruments measuring the same constructs that may have been more sensitive. Further, including qualitative data and a mixed method approach might also have added to the understanding of the effects and neutral results of the intervention. As there were a large number of items, we chose not to measure the quality of the relationship between the dyad members. Measuring marital quality would have been interesting to measure at baseline since this may have influenced the way they responded to the intervention.

Subjective self-care behavior was included among study variables. However, it would have strengthened the study to also include objective measures of behavior such as adherence to treatment regimen, nutrition, and physical activity. Finally, a threat to the validity of the study could be that the dyads in the control group might have received joint education and support as they received care at heart failure clinics. However, in the intervention, partners were actively involved as the patient’s equal, as they were treated as a dyad throughout the whole study. Care as usual at the clinics did not include this component.

Not only the partner, but also family and friend can play an important role as a caregivers. However, we chose partners since they provide the majority of the care and are most likely to be experiencing caregiver burden. There is also an advantage to investigating a more homogenous group. Finally, the length of long-term follow-up can always be discussed. However, since heart failure mortality is high and the dyads consisted of frail and elderly persons, it was difficult to follow the dyads longer than 24 months as the number of patient-partner dyads would have been too small. None of the previous studies in the HF population have followed patients and families longer than 12 months [20,23–25].

## Conclusion

Over the 24-month follow-up period, the intervention had no effect on any of the study outcomes, neither in patients or partners. Considering the fact that family caregivers serve as a critical extension of the formal healthcare system, and that both patients and partners ask for more support, it will become crucial to find new ways to support families affected by heart failure. Contemporary care has remained patient-focused, and there are only a few randomized studies evaluating the effects of dyad interventions. This study may be viewed as a first step in trying to understand dyads’ need for supportive care. Individualized and more targeted interventions seem to be necessary to achieve a higher impact on dyad outcomes.

## Supporting Information

S1 File Study protocol Swedish Ethical-version. (PDF)

S2 File Study protocol translation in English. (DOC)

S1 Table CONSORT 2010 Checklist. (DOCX)

## References

[1] McMurray JJ, Adamopoulos S, Anker SD, Auricchio A, Bohm M, Dickstein K, et al (2012) ESC guidelines for the diagnosis and treatment of acute and chronic heart failure 2012: The Task Force for the Diagnosis and Treatment of Acute and Chronic Heart Failure 2012 of the European Society of Cardiology. Developed in collaboration with the Heart Failure Association (HFA) of the ESC. Eur J Heart Fail 14: 803–869. 10.1093/eurjhf/hfs105 22828712

[2] Moser DK, Dickson V, Jaarsma T, Lee C, Stromberg A, Riegel B (2012) Role of self-care in the patient with heart failure. Curr Cardiol Rep 14: 265–275. 10.1007/s11886-012-0267-9 22437374

[3] Riegel B, Moser DK, Anker SD, Appel LJ, Dunbar SB, Grady KL, et al (2009) State of the science: promoting self-care in persons with heart failure: a scientific statement from the American Heart Association. Circulation 120: 1141–1163. 10.1161/CIRCULATIONAHA.109.192628 19720935

[4] van der Wal MH, van Veldhuisen DJ, Veeger NJ, Rutten FH, Jaarsma T (2010) Compliance with non-pharmacological recommendations and outcome in heart failure patients. Eur Heart J 31: 1486–1493. 10.1093/eurheartj/ehq091 20436049

[5] Clark AP, McDougall G (2006) Cognitive impairment in heart failure. Dimens Crit Care Nurs 25: 93–100; quiz 101–102. 16721180 10.1097/00003465-200605000-00001

[6] Pressler SJ, Subramanian U, Kareken D, Perkins SM, Gradus-Pizlo I, Sauve MJ, et al (2010) Cognitive deficits and health-related quality of life in chronic heart failure. J Cardiovasc Nurs 25: 189–198. 10.1097/JCN.0b013e3181ca36fe 20357665 PMC2922930

[7] Riegel B, Bennett JA, Davis A, Carlson B, Montague J, Robin H, et al (2002) Cognitive impairment in heart failure: issues of measurement and etiology. Am J Crit Care 11: 520–528. 12425402

[8] Buck HG, Harkness K, Wion R, Carroll SL, Cosman T, Kaasalainen S, et al (2014) Caregivers' contributions to heart failure self-care: A systematic review. Eur J Cardiovasc Nurs. 10.1177/1474515113518434 24399843

[9] Gallagher R, Luttik ML, Jaarsma T (2011) Social support and self-care in heart failure. J Cardiovasc Nurs 26: 439–445. 10.1097/JCN.0b013e31820984e1 21372734

[10] Sayers SL, Riegel B, Pawlowski S, Coyne JC, Samaha FF (2008) Social support and self-care of patients with heart failure. Ann Behav Med 35: 70–79. 10.1007/s12160-007-9003-x 18347906

[11] Sebern M, Riegel B (2009) Contributions of supportive relationships to heart failure self-care. Eur J Cardiovasc Nurs 8: 97–104. 10.1016/j.ejcnurse.2008.07.004 18706865

[12] Rosland AM, Piette JD (2010) Emerging models for mobilizing family support for chronic disease management: a structured review. Chronic Illn 6: 7–21. 10.1177/1742395309352254 20308347 PMC4349200

[13] Mårtensson J, Dracup K, Canary C, Fridlund B (2003) Living with heart failure: depression and quality of life in patients and spouses. J Heart Lung Transplant 22: 460–467. 12681424 10.1016/s1053-2498(02)00818-5

[14] Etters L, Goodall D, Harrison BE (2008) Caregiver burden among dementia patient caregivers: a review of the literature. J Am Acad Nurse Pract 20: 423–428. 10.1111/j.1745-7599.2008.00342.x 18786017

[15] Rigby H, Gubitz G, Phillips S (2009) A systematic review of caregiver burden following stroke. Int J Stroke 4: 285–292. 10.1111/j.1747-4949.2009.00289.x 19689757

[16] Molloy GJ, Johnston DW, Witham MD (2005) Family caregiving and congestive heart failure. Review and analysis. Eur J Heart Fail 7: 592–603. 15921800 10.1016/j.ejheart.2004.07.008

[17] Luttik ML, Jaarsma T, Veeger NJ, van Veldhuisen DJ (2005) For better and for worse: Quality of life impaired in HF patients as well as in their partners. Eur J Cardiovasc Nurs 4: 11–14. 15718187 10.1016/j.ejcnurse.2004.12.002

[18] Ågren S, Evangelista L, Davidson T, Stromberg A (2010) The Influence of Chronic Heart Failure in Patient-Partner Dyads-A Comparative Study Addressing Issues of Health-Related Quality of Life. J Cardiovasc Nurs. 10.1097/JCN.0b013e3181ec0281 PMC3246077 21127426

[19] Saunders MM (2003) Family caregivers need support with heart failure patients. Holist Nurs Pract 17: 136–142. 12784897 10.1097/00004650-200305000-00004

[20] Dunbar SB, Clark PC, Deaton C, Smith AL, De AK, O'Brien MC (2005) Family education and support interventions in heart failure: a pilot study. Nurs Res 54: 158–166. 15897791 10.1097/00006199-200505000-00003

[21] Martire LM, Lustig AP, Schulz R, Miller GE, Helgeson VS (2004) Is it beneficial to involve a family member? A meta-analysis of psychosocial interventions for chronic illness. Health Psychol 23: 599–611. 15546228 10.1037/0278-6133.23.6.599

[22] Reid J, Ski CF, Thompson DR (2013) Psychological interventions for patients with coronary heart disease and their partners: a systematic review. PLoS One 8: e73459 10.1371/journal.pone.0073459 24039950 PMC3764157

[23] Sebern MD, Woda A (2012) Shared care dyadic intervention: outcome patterns for heart failure care partners. West J Nurs Res 34: 289–316. 10.1177/0193945911399088 21383082

[24] Lofvenmark C, Saboonchi F, Edner M, Billing E, Mattiasson AC (2013) Evaluation of an educational programme for family members of patients living with heart failure: a randomised controlled trial. J Clin Nurs 22: 115–126. 10.1111/j.1365-2702.2012.04201.x 22946864

[25] Dunbar SB, Clark PC, Reilly CM, Gary RA, Smith A, McCarty F, et al (2013) A trial of family partnership and education interventions in heart failure. J Card Fail 19: 829–841. 10.1016/j.cardfail.2013.10.007 24331203 PMC3869235

[26] Ågren S, Evangelista LS, Hjelm C, Strömberg A (2012) Dyads affected by chronic heart failure: a randomized study evaluating effects of education and psychosocial support to patients with heart failure and their partners. J Card Fail 18: 359–366. 10.1016/j.cardfail.2012.01.014 22555264 PMC3381875

[27] Dickstein K, Cohen-Solal A, Filippatos G, McMurray JJ, Ponikowski P, Poole-Wilson PA, et al (2008) ESC Guidelines for the diagnosis and treatment of acute and chronic heart failure 2008: the Task Force for the Diagnosis and Treatment of Acute and Chronic Heart Failure 2008 of the European Society of Cardiology. Eur Heart J 29: 2388–2442. 10.1093/eurheartj/ehn309 18799522

[28] Strömberg A, Dahlström U, Fridlund B (2006) Computer-based education for patients with chronic heart failure. A randomised, controlled, multicentre trial of the effects on knowledge, compliance and quality of life. Patient Educ Couns 64: 128–135. 16469469 10.1016/j.pec.2005.12.007

[29] Strömberg A, Mårtensson J, Fridlund B, Levin LA, Karlsson JE, Dahlström U (2003) Nurse-led heart failure clinics improve survival and self-care behaviour in patients with heart failure: results from a prospective, randomised trial. Eur Heart J 24: 1014–1023. 12788301 10.1016/s0195-668x(03)00112-x

[30] Stuifbergen A, Becker H, Rogers S, Timmerman G, Kullberg V (1999) Promoting wellness for women with multiple sclerosis. J Neurosci Nurs 31: 73–79. 14964606 10.1097/01376517-199904000-00003

[31] Stuifbergen AK, Becker H, Blozis S, Timmerman G, Kullberg V (2003) A randomized clinical trial of a wellness intervention for women with multiple sclerosis. Arch Phys Med Rehabil 84: 467–476. 12690582 10.1053/apmr.2003.50028

[32] Ware JE, Snow KK, Kosinski M, G B. (1993) SF-36® Health Survey Manual and Interpretation Guide. The Health Institute, Boston: New England Medical Center.

[33] Sullivan M, Karlsson J, Ware JE Jr (1995) The Swedish SF-36 Health Survey—I. Evaluation of data quality, scaling assumptions, reliability and construct validity across general populations in Sweden. Social Science & Medicine 41: 1349–1358. 8560302 10.1016/0277-9536(95)00125-q

[34] Beck AT, Ward CH, Mendelson M, Mock J, Erbaugh J (1961) An inventory for measuring depression. Arch Gen Psychiatry 4: 561–571. 13688369 10.1001/archpsyc.1961.01710120031004

[35] Moser DK, Riegel B, McKinley S, Doering LV, Meischke H, Heo S, et al (2009) The Control Attitudes Scale-Revised: psychometric evaluation in three groups of patients with cardiac illness. Nurs Res 58: 42–51. 10.1097/NNR.0b013e3181900ca0 19092554 PMC2668922

[36] Moser DK, Dracup K (1995) Psychosocial recovery from a cardiac event: the influence of perceived control. Heart Lung 24: 273–280. 7591794 10.1016/s0147-9563(05)80070-6

[37] Årestedt K, Ågren S, Flemme I, Moser D, Strömberg A (2010) Oral Psychometric properties of the Swedish version of the Control Attitudes Scale for patients with cardiac disease and their family members. Eur J Cardiovasc Nurs 9: S26–S27. 10.1177/1474515114529685 24671774

[38] Altman DG (1991) Practical statistics for medical research. London: Chapman and Hall.

[39] Cohen J (2003) Applied multiple regression/correlation analysis for the behavioral sciences. Mahwah, N.J.: L. Erlbaum Associates.

[40] Schaufelberger M, Swedberg K, Koster M, Rosen M, Rosengren A (2004) Decreasing one-year mortality and hospitalization rates for heart failure in Sweden; Data from the Swedish Hospital Discharge Registry 1988 to 2000. Eur Heart J 25: 300–307. 14984918 10.1016/j.ehj.2003.12.012

[41] Peters-Klimm F, Campbell S, Hermann K, Kunz CU, Muller-Tasch T, Szecsenyi J (2010) Case management for patients with chronic systolic heart failure in primary care: the HICMan exploratory randomised controlled trial. Trials 11: 56 10.1186/1745-6215-11-56 20478035 PMC2882359

[42] Ekman I, Wolf A, Olsson LE, Taft C, Dudas K, Schaufelberger M, et al (2012) Effects of person-centred care in patients with chronic heart failure: the PCC-HF study. Eur Heart J 33: 1112–1119. 10.1093/eurheartj/ehr306 21926072 PMC3751966

[43] Jaarsma T, Halfens R, Huijer Abu-Saad H, Dracup K, Gorgels T, van Ree J, et al (1999) Effects of education and support on self-care and resource utilization in patients with heart failure. Eur Heart J 20: 673–682. 10208788 10.1053/euhj.1998.1341

[44] Martire LM, Schulz R, Helgeson VS, Small BJ, Saghafi EM (2010) Review and meta-analysis of couple-oriented interventions for chronic illness. Ann Behav Med 40: 325–342. 10.1007/s12160-010-9216-2 20697859 PMC4101802

[45] Martire LM, Schulz R, Keefe FJ, Rudy TE, Starz TW (2008) Couple-Oriented Education and Support Intervention for Osteoarthritis: Effects on Spouses' Support and Responses to Patient Pain. Fam Syst Health 26: 185–195. 19946460 10.1037/1091-7527.26.2.185 PMC2783596

[46] Birnie K, Garland SN, Carlson LE (2010) Psychological benefits for cancer patients and their partners participating in mindfulness-based stress reduction (MBSR). Psychooncology 19: 1004–1009. 10.1002/pon.1651 19918956

[47] Liljeroos M, Agren S, Jaarsma T, Stromberg A (2014) Perceived caring needs in patient-partner dyads affected by heart failure: A qualitative study. J Clin Nurs. 10.1111/jocn.12588 24698101

[48] Cheng HY, Chair SY, Chau JP (2014) The effectiveness of psychosocial interventions for stroke family caregivers and stroke survivors: A systematic review and meta-analysis. Patient Educ Couns. 10.1016/j.pec.2014.01.005 24485756

[49] Pitteri F, Soulas T, Essertaise AL, Roux J (2013) Contribution of relaxation to a psychoeducational intervention program for family carers of persons with Alzheimer's disease. Geriatr Psychol Neuropsychiatr Vieil 11: 443–451. 10.1684/pnv.2013.0426 24333824

[50] Quinn C, Dunbar SB, Clark PC, Strickland OL (2010) Challenges and strategies of dyad research: cardiovascular examples. Applied Nursing Research 23: e15–e20. 10.1016/j.apnr.2008.10.001 20420989 PMC2861299

[51] Woltz PC, Chapa DW, Friedmann E, Son H, Akintade B, Thomas SA (2012) Effects of interventions on depression in heart failure: a systematic review. Heart Lung 41: 469–483. 10.1016/j.hrtlng.2012.06.002 22938627

[52] Craig P, Dieppe P, Macintyre S, Michie S, Nazareth I, Petticrew M (2008) Developing and evaluating complex interventions: the new Medical Research Council guidance. BMJ 337: a1655 10.1136/bmj.a1655 18824488 PMC2769032

