# Supplementary material for: Long Term Follow-Up after a Randomized Integrated Educational and Psychosocial Intervention in Patient-Partner Dyads Affected by Heart Failure
Source: PLoS One. 2015 Sep 25;10(9):e0138058. doi: 10.1371/journal.pone.0138058 (PMC4583392; doi:10.1371/journal.pone.0138058)
Supplement: S1 File — (PDF) [file pone.0138058.s001.pdf]

HÄLSOUNIVERSITETET
LINKÖPINGS UNIVERSITET

PROTOKOLLsutdrag
Sammanträdesdag
2003-12-16

Närvarande:	Ledamöter	
	B Nordenskjöld	prof, ordförande
	L Bergholtz	doc, vetenskaplig sekr
	J Ahlner	prof, vetenskaplig sekr
	L Bogren	öl
	J Carstensen	prof
	B Lisander	prof
	G Olaison	doc
	U Samuelsson	öl
	K Söderlind	öl, fr o m punkt 4
Repr H-län:	L Brudin	prof
Repr F-län:	S Mölstad	doc
	Lekmannarepresentanter	
	M Tollén	landstingsledamot
	R Wilhelmsson	landstingsledamot
Övriga:	A Alexandersson, adm sekr	
	D Nilsson, psykolog	p 3a
	T Eriksson, spec läk	p 3b

PUNKT	ÄRENDE	BESLUT, ÅTGÄRD

81. Ansökan från Anna Strömberg, Kardiologiska kliniken, Universitetssjukhuset, Linköping: "Effekter av ett vårdprogram med datorbaserad kognitiv, psykosocial och beteendeförändrande terapi till äldre hjärtsviktspatienter och deras anhöriga." Dnr **03-568**

Forskningsetikkommittén beslöt godkänna ansökan.

Vid protokollet — Justeras:

Anna Alexandersson	Lars Bergholtz	Bo Nordenskjöld	John Carstensen
Adm sekr	Docent, vet sekr	Professor, ordförande	Professor

Rätt avskrivet ur protokollet intygas:

Anna Alexandersson

Anna Alexandersson, adm sekr

Forskningsetikkommittén

Postadress:
Forskningsetikkommittén
Klinisk farmakologi
Hälsouniversitetet
581 85 LINKÖPING

Telefon:
013-22 17 45

Telefax:
013-10 41 95

Besöksadress:
Universitetssjukhuset

EPN
Regionala etikprövningsnämnden i Linköping
Avdelningen för prövning av medicinsk forskning

BESLUT

2011-01-26

Anna Strömberg
Avd för omvårdnad/IMH
Linköpings universitet
581 85 LINKÖPING

Ansökan: Effekter av ett vårdprogram med datorbaserad kognitiv, psykosocial och beteendeförändrande terapi till äldre hjärtsviktspatienter och deras anhöriga.
Sökande forskningshuvudman: Linköpings universitet
Dnr **2011/24-32**

Nämnden har erhållit ansökan om ändring av tidigare godkänd ansökan. Ändringen beskrivs i brev daterat 2011-01-19. Ansökan godkänns.

På nämndens vägnar

Staffan Hägg, professor
Vetenskaplig sekreterare

Adress:
Regionala etikprövningsnämnden i Linköping
c/o Hälsouniversitetets kansli
Sandbäcksgatan 7
581 83 LINKÖPING
www.epn.se

Kontaktpersoner:	Telefon:	Fax:
Adm sekr Anna Alexandersson	010 – 103 70 30	013 – 10 44 95
E-post: anna.alexandersson@linkoping.epn.se		
Adm sekr Ewa Westny	010 – 103 41 42	
E-post: ewa.westny@linkoping.epn.se		

Denna ansökningsblankett är gemensam för alla medicinska forskningsetikkommittéer i Sverige. Ansökan skall ifyllas så att den blir lättläst (d v s ej handskrivet och ej med för liten text).

Ankomstdatum ________ Dnr ________

Till forskningsetikkommittén vid Hälsouniversitetet i Linköping

Härmed anhålles om prövning av nedan angivna forskningsprojekt innefattande humanförsök, registerstudie eller liknande:

Linköping — Ort; 031124 — Datum	Undersökningen har granskats och godkänts ur patientsäkerhets- och resurssynpunkter av undertecknad(e) verksamhetschef(er)[1]. *OBS: härmed avses samtliga involverade kliniker/institutioner!*
Anna Strömberg (signature) — Namnteckning (huvudsök./projektansv.)	(signature) — Namnteckning/ar (verksamhetschef eller motsv.)
Anna Strömberg, sjuksköterska, med dr, lektor — Namnförtydligande, titel och tjänst	Eva Swahn, docent, chefsöverläkare — Namnförtydligande, tjänst
Kardiologiska kliniken, US — Institution/klinik	Kardiologiska kliniken, Universitetssjukhuset i Linköping — Plats för undersökningen - ange klinik/institution och adress

581 85 Linköping
Postadress

annst@imv.liu.se
Tel, fax eller email där sökanden kan nås för kompletteringar

	Ja	Nej
Multicenterstudie[2]: (Multicenterprojekt behandlas som **en** ansökan för hela studien!)	☐	☒
Registerstudie:	☐	☒
Affärssekretess viktig att beakta[3]:	☐	☒
Avser upprättande av biobank[4]:	☐	☒

Uppdragsforskning Org.nr: ________

Ansökan avser extern uppdragsforskning[5]: ☐ Ja ☒ Nej
(Om ja, ange uppdragsgivarens adress och kontaktperson)

Jag är medveten om att jag som huvudsökanden vid uppdragsforskning har samma ansvar för forskningsetiken enl. Helsingforsdeklarationen som vid annan forskning (se ssk art. 27 och anvisningen p.5) ☒ Ja

1. Medarbetare (namn, titel och tjänst, arbetsplats - vid omfattande multicenterstudier separat lista, med adresser):
Susanna Ågren, Sjuksköterska, magister i omvårdnad, thoraxintensiven, US i Linköping
Ulf Dahlström, Professor, överläkare, Kardiologiska kliniken, US i Linköping
Jan Mårtensson, sjuksköterska, lektor, Högskola i Halmstad

2. Projekttitel (ge en beskrivande titel på svenska för lekmän, utan sekretesskänslig information):
Effekter av ett vårdprogram med datorbaserad kognitiv, psykosocial och beteendeförändrande terapi till äldre hjärtsviktspatienter och deras anhöriga.

3. Bilagor som bifogas ansökan:

	Ja	Nej
Skriftlig patient/försökspersonsinformation (*skall* i normalfallet bifogas) daterad version	☒	☐
För fackman avsedd detaljerad information (komplettering till punkt 5)	☒	☐
Prövningsplan (daterad eller version nr: ________)	☐	☒
Enkät/intervjuformulär (antal olika formulär: ________)	☒	☐
PUL-anmälan till registeransvarig (vanligtvis personuppgiftsombud)	☐	☒

Övriga bilagor: ________

4. Anhållan om tillstånd eller anmälan har insänts till/inhämtas av:

- ☐ Läkemedelsverket (läkemedelsprövning)[6] Datum: ______
- ☐ Socialstyrelsen (bl a prövning av medicinteknisk produkt)[7] Datum: ______
- ☐ Strålskyddskommittén[8] ______ Datum: ______
- ☐ Datainspektionen[9] Datum: ______
- ☐ Riksarkivet Datum: ______
- ☐ Övrig instans ______ Datum: ______

5a. Sammanfattning av forskningsprogrammet. *Beskrivningen **skall** kunna förstås av kommitténs lekmän! OBS! ansökan **återremitteras** för omskrivning om detta ej beaktas. Undvik terminologi som kräver specialkunskaper. Ange bakgrund, vetenskaplig frågeställning, betydelse av försöksresultaten och motiv för studien (försöksproceduren beskrivs senare, under punkt 9). Beskriv ev. läkemedels effekter och biverkningar kortfattat. För fackmän avsedd detaljerad information kan bifogas som bilaga (förtecknas då under punkt3).*

Antalet personer som lider av hjärtsvikt ökar i hela västvärlden, som en följd av förbättrad medicinsk behandling av hjärtsjukdom och en ökad medellivslängd. En viktig del av hjärtsviktsvården är patientinformation.
Den nya teknologin med multimedia öppnar nya vägar för att göra patientinformation som är interaktiv och styrs av vad patienten och de anhöriga själva är intresserade av att lära sig. Forskning inom andra kroniska sjukdomar framförallt diabetes har visat att interaktiv datorbaserad ökar patienternas kunskaper mer än traditionell patientinformation och att hög ålder och avsaknad av datorerfarenhet inte är ett hinder för att använda datorbaserad utbildning. Studier av effekter för hjärtsviktpatienter och deras anhöriga saknas. Syftet med den här studien är därför att utvärdera effekten av datorbaserad egenvårdsutbildning och psykosocialt stöd till patienter med hjärtsvikt och deras anhöriga med avseende på vårdkonsumtion, överlevnad, BNP, egenvårdsbeteende, upplevd kontroll och börda, livskvalitet samt kostnadseffektivitet.
Metod: Prospektiv, öppen, randomiserad studie där interventionsgruppens patienter och anhöriga får upprepad interaktiv utbildning via CD-ROM om hjärtsvikt vid tre utbildningstillfällen under 3 månader. Vid andra utbildningstillfället träffar patienten en sjuksköterska som bedömer patientens fortsatta behov av utbildning och psykosocialt stöd. Initialt planeras en pilotstudie på 40 patienter som sjukhusvårdats pga hjärtsvikt för att sedan kunna göra powerberäkning för slutgiltigt antal patienter i huvudstudien.
Förväntade resultat: En datorbaserad utbildning för patienter och anhöriga förväntas förbättra patienternas egenvård, livskvalitet och känsla av kontroll och därmed minska bördan på anhöriga, sjuklighet och behovet av vård.
För mer utförlig projektplan se bilaga 1.

5b. Vilken primär vetenskaplig frågeställning ligger till grund för projektets planering? *Ange även eventuella sekundära hypoteser som prövas.*

Utvärdera effekterna av datorbaserad egenvårdsutbildning och psykosocialt stöd till patienter med hjärtsvikt och deras anhöriga med avseende på vårdkonsumtion, överlevnad, BNP, egenvårdsbeteende, upplevd kontroll och börda, livskvalitet samt kostnadseffektivitet.

6. Forskningsetiska överväganden: *Identifiera och precisera vilka etiska problem som kan föreligga. Ange vilka kunskapsvinster studien kan förväntas ge och betydelsen av dessa, vilka risker för skador och obehag som olika aktörer och berörda kan utsättas för, samt ge Din egen värdering av risk-nytto-förhållandet.* ***OBS! Detta är en obligatorisk uppgift.***

Studien planeras och genomförs i enlighet med Helsingfors deklarationen. Innan patienten tar ställning till ev studie inklusion ges muntlig och skriftlig patientinformation. All datainsamling sker först efter skriftligt medgivande av patienten. Alla patienter kommer att få muntlig information om hjärtsvikt. Det finns idag inga riktlinjer för hur utbildning till patient och anhöriga ska ges vid hjärtsvikt och datorutbildning tillhör inte standardvård.

7. Försöksobjekt:

Friska försökspersoner (antal): ______

Patienter (antal): minst 70, max 200 se punkt 8

(vid multicenterstudie anges **både** totalt antal och minimiantal/center)

Om barn, dementa eller personer som av andra skäl ej är kapabla att ge eget informerat samtycke skall ingå, skall detta motiveras under punkt 6

8. Redogör för det statistiska underlaget för patient/försöksmaterialens storlek. Med detta avses en statistisk "power"-beräkning eller motsvarande överväganden.

Eftersom ingen studie av denna typ tidigare gjorts planeras initialt en pilotstudie på 40 patienter som sjukhusvårdats pga hjärtsvikt för att sedan kunna göra en samplesize beräkning för slutgiltigt antal patienter i huvudstudien.

9. Redogör för undersökningsprocedur och resultatinsamling. *Av beskrivningen skall framgå konkret hur studien avses genomföras, d v s typer av ingrepp, mätmetoder, antal besök, tidsåtgång för varje försök, doser och administrationssätt för ev. läkemedel och/eller isotoper, blodprovsmängd (även ackumulerad mängd vid multipla försök). Bifoga gärna flödesscheman eller bilaga med mer fullständig förklaring, så att kommittén lätt kan förstå hur försöken går till. Sammanfatta dock alltid den viktigaste informationen nedan.*

Datainsamling av demografiska data sker via ett standardiserat formulär utan identifierande patientuppgifter. En del data samlas in från patientjournaler. BNP sker via venprovtagning.

Livskvalitet

Mätning av den hälsorelaterade livskvaliten görs med ett livkvalitetsformulär specifikt för hjärtsvikt: Minnesota Living with Heart Failure Questionnaire och ett allmänt livskvalitetsformulär: SF 36

Egenvård mäts med ett instrument som heter "the European Self-care Behaviour Scale". Upplevd Börda mäts med The Caregiver's Burden Scale (CBS).Upplevd kontroll mäts med 4 frågor.

Antal vårdbesök, återinläggningar och vårddagar på sjukhus

Dataformulär ifylles med hjälp av dokumentation i journalen och uppgifter från patienten angående antal återinläggningar på sjukhus, antal vårddagar samt antal polikliniska besök på sjukhus och vårdcentral.

Hälsoekonomi: kostnaden för de olika modellerna

Hälsoekonomiska beräkningar: att sätta prislappar på alla delar som ingår i intervention respektive kontroll grupp och beräkna genomsnittskostnad för varje patient.

Tre olika mättillfällen. Det första genomförs före utbildningen i samband med besök på hjärtsviktsmottagningen eller inneliggande på hjärtsviktsavdelning och de övriga två vid månad 3 och 12. Sjukvårdskonsumtion och överlevnad mäts genom journalgranskning efter 12 månader.

Mättillfälle 1: Demografiska data, medicinskt status, BNP, egenvårdsbeteende, upplevd kontroll och börda och livskvalitet vid första besöket.

Mättillfälle 2: Medicinskt status, BNP, egenvårdsbeteende, upplevd kontroll och börda och livskvalitet 3 månader efter inklusion.

Mättillfälle 3: Medicinskt status, BNP, egenvårdsbeteende, upplevd kontroll och börda och livskvalitet samt mätning av sjukvårdskonsumtion och överlevnad genom journalgranskning efter 12 månader.

10. Redogör för komplikationer (även smärta, obehag eller integritetsintrång). *Ange även åtgärder för att förebygga och/eller behandla dessa.*

Inga kan förutses eller förväntas.

11. Redogör för resultat från relevanta djurförsök. *Om djurförsöket ej utförts, ange skälen till att initialt utföra humanförsök.*

–

12. Redogör för tidigare erfarenheter (egna och/eller andra) av den använda tekniken eller behandlingen. *Om ansökan avser fortsättning på/uppföljning av tidigare godkänt projekt, ange Dnr och beslutsdatum för tidigare godkänd ansökan. Om tekniken enbart använts i djurförsök bör även dessa erfarenheter redovisas. Vid nya behandlingar inkluderande läkemedel bör anges hur många patienter (med aktuell, respektive annan åkomma), som tidigare erhållit föreslagen och/eller högre dosering, samt hur långa behandlingsperioder som tidigare studerats.*

Alla medarbetare har lång erfarenhet av handläggning av hjärtpatienter samt av att genomföra randomiserade studier.

Studier som utvärderar effekter av en omfattande datorbaserad utbildning till patienter med hjärtsvikt och deras anhöriga saknas.

13. Ange relation mellan försöksledare och patient/försöksperson.[11]

☐ Läkare-patient ☐ Kursgivare-student ☐ Arbetsgivare-anställd

☒ Annan: sjuksköterska (ej omvårdnadsansvarig) - patient

14. Hur utväljes patienter/försökspersoner? *Vid annonsering efter patienter och/eller försökspersoner skall annonsen tillställas kommittén för godkännande. Annonsering bör överensstämma med den skriftliga informationen enligt punkt 18, även om annonsen är mer koncis. Ange om patienter/försökspersoner rekryterats från tidigare eller pågående studier. Ange inklusions- och exklusionskriterier.*

Patienter som sjukhusvårdats pga hjärtsvikt och deras anhöriga tillfrågas om deltagande i studien.

15. Kan patienterna/försökspersonerna komma att inkluderas i flera studier samtidigt eller i nära anslutning till denna studie? *Om så är fallet ange projekttitel, ansvarig, samt Dnr för de andra studierna.*[13]

Nej

16. Ange formerna för och registrering av hälsokontroll av friska försökspersoner.

–

17. Redogör för registreringen av resultat och ev. komplikationer. *Ange formerna för registrering i form av försöksprotokoll och journalanteckningar. Bifoga ev. intervjuformulär eller enkäter och formulär för registrering av resultat. Ange sekretesskydd vid t ex datorbearbetning av resultat eller videoinspelning. Data bör vanligen vara kodade vid bearbetning.*

Endast den sjukvårdspersonal som fyller i formulären och följer upp patienten kan identifiera en bestämd patient med ett bestämt formulär. Det signerade patientmedgivandet kommer att förvaras i patientens journal. Kopia på patientmedgivandet tilldelas patienten.
Studien kommer att sammanställas så att enskilda personuppgifter inte kan kopplas samman med enskilda fall. All data kommer att avidentifieras.
Alla instrument bifogas i bilaga 2.

18. Hur informeras patienterna/försökspersonerna och hur inhämtas samtycke? *Förutom den muntliga informationen skall som regel en kortfattad och lättförståelig* ***skriftlig*** *information ges! Denna skall bifogas ansökan. Om avsikten är att ge endast muntlig information, beskrives i denna ruta eller på bilaga innehållet i den muntliga informationen. Om ingen eller ofullständig information ges, måste detta noga motiveras! Se förklaring "för ytterligare upplysningar"!* [12]

Skriftlig och muntlig information om studien ges till patienten och dennes make/maka, se bilaga 3 under sjukhusvistelsen. Patienten och dennes anhörige tar sedan ställning till deltagande och lämnar vid deltagande ett skriftligt samtycke. Skriftlig information bifogas i bilaga 3.

19. Vilka ekonomiska ersättningar eller andra förmåner utgår till deltagarna i projektet?

Ersättning för "sveda och värk". Ange ev. belopp (före skatt); ____________

Ersättning för förlorad arbetsinkomst (om tillämpligt)	☐ Ja	☒ Nej
Reseersättning	☐ Ja	☒ Nej
Befrielser från kostnader för läkemedel (om tilllämpligt)	☐ Ja	☒ Nej

Befrielser från andra kostnader i samband med sjukvård, eller andra förmåner?: ____________

20. Vilket försäkringsskydd finns för deltagarna i projektet? *Det åligger projektledaren att kontrollera om befintliga försäkringar täcker skador som kan uppkomma. Vid behov tecknas speciell försäkring.*

Sedvanlig patientförsäkringen

Projektplan

1. Titel
Effekter av ett vårdprogram med datorbaserad kognitiv, psykosocial och beteendeförändrande terapi till äldre hjärtsviktspatienter och deras anhöriga.

2. Bakgrund

Antalet personer som lider av hjärtsvikt ökar i hela västvärlden, som en följd av minskad mortalitet vid akut hjärtinfarkt, förbättrad medicinsk behandling av hjärtsvikt och en ökad medellivslängd.[1] I Sverige har c:a 200 000 individer symtomgivande hjärtsvikt. Både incidens och prevalens är starkt åldersberoende. Hjärtsvikt är den vanligaste orsaken till sjukhusinläggning bland personer över 65 år och hjärtsviktsvården konsumerar årligen ca 1-2% av den totala sjukvårdsbudgeten. Sjukhuskostnader svarar för mellan 50 och 75% av de totala sjukvårdskostnaderna för hjärtsvikt och hjärtsviktspatienterna står för 30% av alla vårddagar som orsakas av hjärtsjukdom.[2] En stor del av sjukhusinläggningarna orsakas inte av den underliggande sjukdomen utan av andra faktorer som är möjliga att förebygga. Flera studier har visat att ca hälften av alla inläggningar troligtvis kunde ha förhindrats. Bidragande faktorer till de inläggningar som kan förebyggas är framförallt att patienten ej följer läkemedelsordinationer, livsstil- och kostråd.[3] För att hjärtsviktspatienternas hälsa och prognos ska förbättras och behovet av sjukhusvård minska är det nödvändigt att den föreskrivna behandlingen följs. Information till patienterna och deras anhöriga är viktigt för att motivera till detta. Patientinformation utgör därför idag en viktig del av omvårdnaden och behandlingen av hjärtsviktspatienter. Målet är en patient som har förståelse för sin sjukdom och dess behandling och som delvis kan ta ansvar för enklare kontroller såsom vikt och vätskemängd. En del patienter kan även justera diuretikadoser vid ev försämring.
Det övergripande målet är att få en patient som kan bedriva egenvård och som på ett tidigt stadium tar kontakt med sin sjuksköterska/läkare vid eventuella försämringar, för att undvika att sjukdomsförloppet blir så allvarligt att patienten måste uppsöka akuten och/eller läggas in. Utvecklingen av patientutbildning till patienter med hjärtsvikt har utvecklats under det senaste decenniet. Det finns studier som utvärderar patientutbildning i kombination med intensifierad uppföljning och optimerad behandling. Men studier som enbart utvärderar effekten av utbildning är få.[4]
Den nya teknologin med multimedia öppnar nya vägar för att göra patientinformation som är interaktiv och styrs av vad patienten och de anhöriga själva är intresserade av att lära sig. Forskning inom andra kroniska sjukdomar framförallt diabetes har visat att interaktiv datorbaserad ökar patienternas kunskaper mer än traditionell patientinformation och att hög ålder och avsaknad av datorerfarenhet inte är ett hinder för att använda datorbaserad utbildning. Studier av effekter för anhöriga saknas.

3. Syfte

Att utvärdera effekten av datorbaserad egenvårdsutbildning och psykosocialt stöd till patienter med hjärtsvikt och deras anhöriga med avseende på vårdkonsumtion, överlevnad, BNP, egenvårdsbeteende, upplevd kontroll och börda, livskvalitet samt kostnadseffektivitet.

4. Hypotes

Datorbaserad egenvårdsutbildning och psykosocialt stöd till patienter med hjärtsvikt och deras anhöriga förbättrar patienternas egenvårdbeteende, livskvalitet och känsla av kontroll och minskar därmed sjuklighet och behovet av vård.

5. Metod

5:1 Design
Prospektiv, öppen, randomiserad studie med utvärdering av interaktiv datorbaserad egenvårdsutbildning och psykosocialt stöd till patienter med hjärtsvikt och deras anhöriga.

5:2 Interventionen
Interventionsgruppens patienter och anhöriga får upprepad interaktiv utbildning via CD-ROM om hjärtsvikt vid minst tre utbildningstillfällen under en period av 2-3 månader. Vid andra utbildningstillfället träffar patienten en sjuksköterska som bedömer patientens fortsatta behov av utbildning och psykosocialt stöd.

5:3 Patienter
Män och kvinnor som diagnostiserats med hjärtsvikt enligt European Society of Cardiology:s riktlinjer. [5]
Initialt planeras en pilotstudie på 50 patienter som sjukhusvårdats pga hjärtsvikt för att sedan kunna göra powerberäkning för slutgiltigt antal patienter i huvudstudien.
Målet är att sedan rekrytera det antal patienter som räknas fram i samplesizeberäkningen från kardiologiska kliniken i Linköping.

5:4 Datainsamling och datahantering
Mätinstrument
Datainsamling av demografiska data sker via ett standardiserat formulär utan identifierande patientuppgifter. En del data samlas in från patientjournaler. BNP sker via venprovtagning.
Livskvalitet
Mätning av den hälsorelaterade livskvaliten görs med ett livkvalitetsformulär specifikt för hjärtsvikt: Minnesota Living with Heart Failure Questionnaire. Frågorna handlar om hur hjärtsvikten påverkar livssituationen. Har hjärtsvikten hindrat personen från att leva som hon/han önskat? [6]
Ett allmänt livskvalitetsformulär: SF 36 kommer också att användas. Frågorna är allmänna och handlar om hur patienten uppfattar sitt hälsotillstånd och ev inskränkningar i livet som orsakas av hälsotillståndet [7].
Egenvård
Egenvård mäts med ett instrument som heter "the European Self-care Behaviour Scale". Instrumentet innehåller 12 olika påståenden om egenvård vid hjärtsvikt. Svarsalternativen beskriver en skala mellan ytterligheterna stämmer precis (1) och stämmer inte alls (5). Instrument är testat och har tillfredställande validitet och reliabilitet. [8]
Upplevd Börda
The Caregiver's Burden Scale (CBS) är en skal med 22 item som subjektivt bedömer vårdarens upplevda börda vid kronisk sjukdom. Varje item har 4 svarsalternativ. Skalan uppdelas i 5 faktorer: allmän , isolering, besvikelse, emotionell påverkan och omgivningen. Instrument är testat och har tillfredställande validitet och reliabilitet. [9]

Upplevd kontroll
Instrument med 4 frågor om upplevd kontroll i olika situationer. Svarsalternativen beskriver en skala mellan ytterligheterna ingen (1) och väldigt mycket (5).
Antal vårdbesök, återinläggningar och vårddagar på sjukhus
Dataformulär ifylles med hjälp av dokumentation i journalen och uppgifter från patienten angående antal återinläggningar på sjukhus, antal vårddagar samt antal polikliniska besök på sjukhus och vårdcentral.
Hälsoekonomi: kostnaden för de olika modellerna
Hälsoekonomiska beräkningar: att sätta prislappar på alla delar som ingår i intervention respektive kontroll grupp och beräkna genomsnittskostnad för varje patient.
Kostnaden kan även jämföras med livskvalitet.

Tre olika mättillfällen enligt nedan. Det första genomförs före utbildningen i samband med besök på hjärtsviktsmottagningen eller inneliggande på hjärtsviktsavdelning och de övriga två vid månad 3 och 12. Sjukvårdskonsumtion och överlevnad mäts genom journalgranskning efter 12 månader.
Mättillfälle 1: Demografiska data, medicinskt status, BNP, egenvårdsbeteende, upplevd kontroll och börda och livskvalitet vid första besöket.
Mättillfälle 2: Medicinskt status, BNP, egenvårdsbeteende, upplevd kontroll och börda och livskvalitet 3 månader efter inklusion.
Mättillfälle 3: Medicinskt status, BNP, egenvårdsbeteende, upplevd kontroll och börda och livskvalitet samt mätning av sjukvårdskonsumtion och överlevnad genom journalgranskning efter 12 månader.

5:4:1 Demografiska och kliniska data:
Kön, ålder, utbildningsnivå, civilstånd, bostad-omvårdnadsform, vårdtillfällen under studietiden, etiologi, medicinsk status, farmakologisk behandling av hjärtsvikt

Endast den sjukvårdspersonal som fyller i formulären och följer upp patienten kan identifiera en bestämd patient med ett bestämt formulär. Det signerade patientmedgivandet kommer att förvaras i patientens journal. Kopia på patientmedgivandet tilldelas patienten.
Studien kommer att sammanställas så att enskilda personuppgifter inte kan kopplas samman med enskilda utfall.

5:5 Databehandling, databearbetning och statistiska analyser
Data kommer direkt att matas in, sammanställas och behandlas i en databas. Inga pappersformulär kommer att användas i studien. Enskilda patienter kommer ej att kunna identifieras i de slutliga resultaten.
Jämförelse mellan grupperna kommer att göras med chi-två för nominala variabler och t-test för normalfördelade kontinuerliga variabler. Mann-Whitney U test kommer att användas för icke normalfördelade kontinuerliga variabler. Parat *t* test och Wilcoxon rangsummetest används för jämförelse inom grupper. Kaplan-Meier kurvor konstrueras för att bedöma skillnad mellan modellerna i antalet överlevande patienter utan sjukhusinläggning under 12 månaders uppföljningstid och log-rank test används för att jämföra skillnader mellan grupperna i överlevnad utan sjukhusinläggning. Alla analyser görs på basen av "intention att behandla". Ett *P*–värde mindre än 0.05 anses som statistiskt signifikant.

6. Betydelse

För att förbättra mortalitet och morbiditet och öka välbefinnande för personer med hjärtsvikt krävs att de bedriver egenvård dvs följer den farmakologiska behandling och livsstilsråd samt lär sig att känna igen och åtgärda symtom på försämring. Målet med patientutbildning och uppföljning är ofta att öka patienternas egenvårdsförmåga, men eftersom majoriteten av patienterna med hjärtsvikt är över 75 år, ofta multisjuka och med kognitiva problem kan det vara svårt. Ett par amerikanska studier som har utgått ifrån patientens perspektiv har funnit att patienterna ofta har svårt att se värdet med egenvård och svårt att tex lära sig att känna igen och tolka symtom. Det saknas Europeiska studier inom området och resultatet. Kan användas till att utveckla utbildning och stöd till patienten utvecklas så att egenvården ökar och morbiditeten och mortaliteten minskar

7. Referenser

[1] Cowie MR, Mosterd A, Wood DA, et al. The epidemiology of heart failure. Eur Heart J 1997;18:208-225.

[2] Ryden-Bergsten T, Andersson F. The health care costs of heart failure in Sweden. J Intern Med 1999;246:275-284.

[3] Evangelista LS, Dracup K. A closer look at compliance research in heart failure patients in the last decade. Prog Cardiovasc Nurs 2000;15:97-103.

[4] Strömberg A. Educating patients and Nurses to manage heart failure better. Eur J Cardiovasc Nurs 2002;1:33-40.

[5] The Task Force of the Working Group on Heart Failure of the European Society of Cardiology. Guidelines for the diagnosis and treatment of chronic heart failure. Eur Heart J 2001;22:1527-1560.

[6] Rector, T.S. Cohn, J.N. Assessment of patient outcome with the Minnesota living with heart failure questionnaire: Reliability and validity during a randomized, double-blind, placebo-controlled trial of pimobendan. Am Heart J 1992;124:1017-27.

[7] Ware JE. SF-36 health survey manual and interpretation guide. Boston: The New Health Institute, New England Medical Center, 1993.

[8] Jaarsma T, Strömberg A, Mårtensson J, Dracup K. Development and testing of the European Heart Failure Self-Care Behaviour Scale. European Journal of Heart Failure 2003;5(3):363-70

[9] Elmståhl S, Malmberg B, Annerstedt L. Caregivers burden of patients 3 years after stroke assessed by a novel Caregiver Burden Scale. Arch Phys Med Rehabil 1996; 77: 177-82.

Bilaga 2

ATT LEVA MED HJÄRTSVIKT - FRÅGEFORMULÄR

Frågorna handlar om i vilken utsträckning Din hjärtsvikt har hindrat Dig från att leva som Du vill den senaste månaden. Varje fråga tar upp ett av de sätt man kan påverkas på. Om Du är säker på att det som sägs i frågan inte gäller Dig eller inte har något samband med Din hjärtsvikt, ringa in nollan (0=nej) och gå vidare till nästa fråga. Om det som sägs i frågan stämmer in på Dig ska Du ringa in den siffra som bäst beskriver hur mycket Du hindrats att leva som Du vill den senaste månaden.

Har Din hjärtsvikt hindrat Dig från att leva som du vill den senaste månaden genom att:

		Nej	Väldigt lite				Väldigt mycket
1	Ha orsakat svullnad i tex vrister och ben?	0	1	2	3	4	5
2	Du har varit tvungen att sätta Dig eller lägga Dig ner för att vila under dagen?	0	1	2	3	4	5
3	Ha gjort det svårt för Dig att gå omkring eller gå i trappor?	0	1	2	3	4	5
4	Ha gjort det svårt för Dig att sköta hushållssysslor eller arbeta i trädgården?	0	1	2	3	4	5
5	Ha gjort det svårt för Dig att komma ut från Ditt hem?	0	1	2	3	4	5
6	Ha gjort det svårt för Dig att sova gott om natten?	0	1	2	3	4	5
7	Ha gjort det svårt för Dig att umgås med eller göra saker tillsammans med vänner och familj?	0	1	2	3	4	5
8	Ha gjort det svårt för Dig att förvärvsarbeta?	0	1	2	3	4	5
9	Ha gjort det svårt för Dig att ägna Dig åt fritidsysselsättningar, idrott eller hobbies?	0	1	2	3	4	5

Bilaga 4

Har Din hjärtsvikt hindrat Dig från att leva som du vill den senaste månaden genom att:

	Nej	Väldigt lite				Väldigt mycket
10 Ha försvårat Ditt sexualliv?	0	1	2	3	4	5
11 Du har varit tvungen att äta mindre av sådant Du tycker om?	0	1	2	3	4	5
12 Ha gjort Dig andfådd?	0	1	2	3	4	5
13 Ha gjort Dig trött, matt eller orkeslös?	0	1	2	3	4	5
14 Du har varit tvungen att ligga på sjukhus?	0	1	2	3	4	5
15 Ha kostat Dig pengar för sjukvård?	0	1	2	3	4	5
16 Du har fått biverkningar av medicinerna?	0	1	2	3	4	5
17 Ha fått Dig att känna Dig som en börda Din familj eller Dina vänner?	0	1	2	3	4	5
18 Ha fått Dig att känna att Du har mindre kontroll över Ditt eget liv?	0	1	2	3	4	5
19 Ha gjort att Du oroar Dig?	0	1	2	3	4	5
20 Ha gjort det svårt för Dig att koncentrera Dig eller komma ihåg saker?	0	1	2	3	4	5
21 Ha gjort Dig ledsen eller nedstämd?	0	1	2	3	4	5

Patientens initialer:______ Patientens nummer:______ Besök: 1 2 3

Datum:____I_____I____ Center:________________

år månad dag

FRÅGEFORMULÄR OM HÄLSA

Detta formulär innehåller frågor om hur DU ser på Din hälsa. Besvara frågorna genom att sätta en ring runt den siffra som Du tycker stämmer bäst in på Dig. Om Du är osäker, ringa ändå in den siffra som känns mest rätt.

1. I allmänhet, skulle Du vilja säga att Din hälsa är:

(sätt en ring runt en siffra)

Utmärkt 1

Mycket god 2

God 3

Någorlunda 4

Dålig 5

2. Jämfört med ett år sedan hur skulle Du vilja bedöma Ditt allmänna tillstånd nu?

(sätt en ring runt en siffra)

Mycket bättre nu än för ett år sedan 1

Något bättre nu än för ett år sedan 2

Ungefär detsamma 3

Något sämre nu än för ett år sedan 4

Mycket sämre nu än för ett år sedan 5

Anhörigs initialer:________ Patientens kod:________ (ifylles av distriktssköterska)

Datum:_____I_____I_____ Center:_______________ (ifylles av distriktssköterska)
år månad dag

Copyright © 1994 Medical Outcomes Trust
All rights reserved.
(IQOLA SF-36 Standard Swedish Version 1.0)

3. De följande frågorna handlar om aktiviteter som Du kan tänkas utföra under en vanlig dag. Är Du på grund av Ditt hälsotillstånd begränsad i dessa aktiviteter nu? Om så är fallet, hur mycket?

(sätt en ring runt en siffra på varje rad)

	**Ja, mycket begränsad**	**Ja, lite begränsad**	**Nej, inte alls begränsad**
a. Ansträngande aktiviteter, som att springa, lyfta tunga saker, delta i ansträngande sporter	1	2	3
b. Måttligt ansträngande aktiviteter, som att flytta ett bord, dammsuga, skogspromenader eller trädgårdsarbete	1	2	3
c. Lyfta eller bära matkassar	1	2	3
d. Gå uppför flera trappor	1	2	3
e. Gå uppför en trappa	1	2	3
f. Böja Dig eller gå ner på knä	1	2	3
g. Gå mer än två kilometer	1	2	3
h. Gå några hundra meter	1	2	3
i. Gå hundra meter	1	2	3
j. Bada eller klä på sig	1	2	3

4. Under de senaste fyra veckorna, har Du haft något av följande problem i Ditt arbete eller med andra regelbundna dagliga aktiviteter som en följd av Ditt kroppsliga hälsotillstånd?

(sätt en ring runt en siffra på varje rad)

	**JA**	**NEJ**
a. Skurit ned den tid Du normalt ägnat åt arbete eller andra aktiviteter	1	2
b. Uträttat mindre än Du skulle önskat	1	2
c. Varit hindrad att utföra vissa arbetsuppgifter eller andra aktiviteter	1	2
d. Haft svårigheter att utföra Ditt arbete eller andra aktiviteter (t.ex. genom att det krävde extra ansträngning)	1	2

5. Under de senaste fyra veckorna, har Du haft något av följande problem i Ditt arbete eller med andra regelbundna dagliga aktiviteter som en följd av känslomässiga problem (som t.ex. nedstämdhet eller ängslan)?

(sätt en ring runt en siffra på varje rad)

	JA	NEJ
a. Skurit ned den tid Du normalt ägnat åt arbete eller andra aktiviteter	1	2
b. Uträttat mindre än Du skulle önskat	1	2
c. Inte utfört arbete eller andra aktiviteter så noggrant som vanligt	1	2

6. Under de senaste fyra veckorna, i vilken utsträckning har Ditt kroppsliga hälsotillstånd eller Dina känslomässiga problem stört Ditt vanliga umgänge med anhöriga, vänner, grannar eller andra?

(sätt en ring runt en siffra)

Inte alls 1

Lite 2

Måttligt 3

Mycket 4

Väldigt mycket 5

7. Hur mycket värk eller smärta har Du haft under de senaste fyra veckorna?

(sätt en ring runt en siffra)

Ingen 1

Mycket lätt 2

Lätt 3

Måttlig 4

Svår 5

Mycket svår 6

8. Under de senaste fyra veckorna, hur mycket har värken eller smärtan, stört Ditt normala arbete (innefattar både arbete utanför hemmet och hushållssysslor)?

(sätt en ring runt en siffra)

Inte alls 1

Lite 2

Måttligt 3

Mycket 4

Väldigt mycket 5

9. Frågorna här handlar om hur Du känner Dig och hur Du haft det under de senaste fyra veckorna. Ange för varje fråga det svarsalternativ som bäst beskriver hur Du känt Dig. Hur stor del av tiden under de senaste fyra veckorna ....

	**Hela tiden**	**Största delen av tiden**	**En hel del av tiden**	**En del av tiden**	**Lite av tiden**	**Inget av tiden**
a. Har Du känt Dig riktigt pigg och stark?	1	2	3	4	5	6
b. Har Du känt Dig mycket nervös?	1	2	3	4	5	6
c. Har Du känt Dig så nedstämd att ingenting kunnat muntra upp dig?	1	2	3	4	5	6
d. Har Du känt Dig lugn och harmonisk?	1	2	3	4	5	6
e. Har Du varit full av energi?	1	2	3	4	5	6
f. Har Du känt Dig dyster och ledsen?	1	2	3	4	5	6
g. Har Du känt Dig utsliten?	1	2	3	4	5	6
h. Har Du känt Dig glad och lycklig?	1	2	3	4	5	6
i. Har Du känt Dig trött?	1	2	3	4	5	6

10. Under de senaste fyra veckorna, hur stor del av tiden har Ditt kroppsliga hälsotillstånd eller Dina känslomässiga problem stört Dina möjligheter att umgås (t.ex. hälsa på släkt, vänner, etc.)?

(sätt en ring runt en siffra)

Hela tiden 1

Största delen av tiden 2

En del av tiden 3

Lite av tiden 4

Inget av tiden 5

11. Välj det svarsaltenativ som bäst beskriver hur mycket vart och ett av följande påståenden STÄMMER eller INTE STÄMMER in på Dig.

	**Stämmer precis**	**Stämmer ganska bra**	**Osäker**	**Stämmer inte särskilt bra**	**Stämmer inte alls**
a. Jag verkar ha lite lättare för att bli sjuk än andra människor	1	2	3	4	5
b. Jag är lika frisk som vem som helst av dem jag känner	1	2	3	4	5
c. Jag tror min hälsa kommer att bli sämre	1	2	3	4	5
d. Min hälsa är utmärkt	1	2	3	4	5

EGENVÅRD VID HJÄRTSVIKT

Detta formulär innehåller påståenden om egenvård vid hjärtsvikt. Besvara varje påstående genom att sätta en ring runt den siffra som Du tycker stämmer bäst in på Dig. Notera att svarsalternativen beskriver en skala mellan ytterligheterna stämmer precis (1) och stämmer inte alls (5). Om Du är osäker, ringa ändå in den siffra som känns mest rätt för Dig.

	Stämmer precis				Stämmer inte alls
1. Jag väger mig varje dag	1	2	3	4	5
2. Om jag blir andfådd tar jag det lugnt	1	2	3	4	5
3. Vid ökad andfåddhet kontaktar jag min läkare eller distriktssköterska	1	2	3	4	5
4. Om mina fötter/ben svullnar mer än vanligt kontaktar jag min läkare eller distriktssköterska	1	2	3	4	5
5. Om jag ökar två kilo i vikt på en vecka kontaktar jag min läkare eller distriktssköterska	1	2	3	4	5
6. Jag begränsar mitt vätskeintag (inte mer än 1,5-2 liter/dygn)	1	2	3	4	5
7. Jag vilar en stund på dagen	1	2	3	4	5
8. Om jag upplever en ökad trötthet kontaktar jag min läkare eller distriktssköterska	1	2	3	4	5
9. Jag äter en kost med lite salt i	1	2	3	4	5
10. Jag äter mina mediciner som läkaren ordinerat	1	2	3	4	5
11. Jag tar en influensa vaccination varje år	1	2	3	4	5
12. Jag motionerar regelbundet	1	2	3	4	5

Patientens initialer:________ Patientens kod:________

Datum:____I____I____ Center:________________
år månad dag

Europeiska beteendeskalan för egenvård vid hjärtsvikt
(Jaarsma, Strömberg, Mårtensson, Dracup, 1999)

Frågeformulär om Upplevd Kontroll

Frågorna handlar om hur mycket kontroll du upplever att du har i olika situationer.
Ringa in den siffra som bäst beskriver ditt svar på följande frågor.

		Ingen		Moderate		Väldigt mycket
1.	Hur mycket kontroll upplever du att du har över ditt liv?	1	2	3	4	5
2.	Hur mycket hjälplöshet känner du relaterat till din sjukdom?	1	2	3	4	5
3.	Känner du oro relaterat till din hälsa?	1	2	3	4	5
4.	Hur mycket oro känner du inför risken att drabbas av en plötslig eller oväntad akut hjärthändelse?	1	2	3	4	5

Anhörigfrågor

1. Känner Du Dig trött och utarbetad?

0	Nej, inte alls
1	Nej, knappast
2	Ja, i viss mån
3	Ja, i hög grad

2. Känner Du Dig ensam och isolerad p g a Din anhöriges problem?

0	Nej, inte alls
1	Nej, knappast
2	Ja, i viss mån
3	Ja, i hög grad

3. Tycker Du att Du får ta för mycket ansvar för Din anhöriges väl och ve?

0	Nej, inte alls
1	Nej, knappast
2	Ja, i viss mån
3	Ja, i hög grad

4. Känns det ibland som om Du skulle vilja fly undan hela den situation Du befinner Dig i?

0	Nej, inte alls
1	Nej, knappast
2	Ja, i viss mån
3	Ja, i hög grad

5. Ställs Du inför rent praktiska problem i vården som Du tycker är svåra att lösa?

0	Nej, inte alls
1	Nej, knappast
2	Ja, i viss mån
3	Ja, i hög grad

6. Kan Du bli sårad och arg på Din anhörige?

0	Nej, inte alls
1	Nej, knappast
2	Ja, i viss mån
3	Ja, i hög grad

7. Tycker Du att Din hälsa har blivit lidande p g a att Du tagit hand om Din anhörige?

0	Nej, inte alls
1	Nej, knappast
2	Ja, i viss mån
3	Ja, i hög grad

8. Har Din anhöriges problem lett till att umgänget med andra, t ex släkt och vänner, minskat för Dig?

0	Nej, inte alls
1	Nej, knappast
2	Ja, i viss mån
3	Ja, i hög grad

9. Finns det något i Din anhöriges bostad som gör det besvärligt att ta hand om honom eller henne?

0	Nej, inte alls
1	Nej, knappast
2	Ja, i viss mån
3	Ja, i hög grad

10. Känner Du Dig bunden av Din anhöriges problem?

0	Nej, inte alls
1	Nej, knappast
2	Ja, i viss mån
3	Ja, i hög grad

11. Känner Du Dig besvärad av Din anhöriges beteende?

0	Nej, inte alls
1	Nej, knappast
2	Ja, i viss mån
3	Ja, i hög grad

12. Leder Din anhöriges problem till att Du inte kan göra det Du hade tänkt göra vid denna tid i livet?

0	Nej, inte alls
1	Nej, knappast
2	Ja, i viss mån
3	Ja, i hög grad

Patientinformation

Effekter av datorbaserad kognitiv, psykosocial och beteende-förändrande terapi till äldre hjärtsviktspatienter och deras anhöriga.

Antalet personer som lider av hjärtsvikt ökar i hela västvärlden, som en följd av förbättrad medicinsk behandling av hjärtsjukdom och en ökad medellivslängd. En viktig del av hjärtsviktsvården är patientinformation. Den nya teknologin med multimedia öppnar nya vägar för att göra patientinformation som är interaktiv och styrs av vad patienten och de anhöriga själva är intresserade av att lära sig. Forskning inom andra kroniska sjukdomar framförallt diabetes har visat att interaktiv datorbaserad ökar patienternas kunskaper mer än traditionell patientinformation och att hög ålder och avsaknad av datorerfarenhet inte är ett hinder för att använda datorbaserad utbildning. Studier av effekter för hjärtsviktpatienter och deras anhöriga saknas. Syftet med den här studien är därför att utvärdera effekten av datorbaserad egenvårdsutbildning och psykosocialt stöd till patienter med hjärtsvikt och deras anhöriga.

Om Du och din anhörig bestämmer Er för att delta kommer Ni enligt en slumpvis uppgjord ordning att antingen få traditionell uppföljning efter sjukhusvård eller en datorbaserad utbildning och psykosocialt stöd från hjärtsviktssjuksköterska. Ni kommer att få svara på fyra enkäter avseende livskvalitet, egenvård, upplevd börda och kontroll. Efter tre och tolv månader kommer Ni att kallas till ett återbesök för uppföljning och provtagning.

Vi kommer också att hämta vissa uppgifter från journalen såsom orsaken till hjärtsvikten. Dessa uppgifter kommer att läggas in i en databas. Uppgifterna är helt anonyma och inga obehöriga kan ta del av uppgifterna.

Er medverkan i undersökningen är helt frivillig och kan avbrytas när som helst utan att det påverkar omhändertagandet eller vården. Alla uppgifter behandlas konfidentiellt och studien är godkänd av Forsknings-etikkommittén vid Hälsouniversitetet i Linköping och av Ewa Svahn, chefsöverläkare vid kardiologiska kliniken, Universitetssjukhuset i Linköping.

Medgivande formulär

Vi intygar härmed att vi erhållit såväl muntlig som skriftlig information om studien "Effekter av datorbaserad kognitiv, psykosocial och beteendeförändrande terapi till äldre hjärtsviktspatienter och deras anhöriga. " och att vi samtycker till att delta. Vi är medveten om att vi när som helst kan avbryta deltagandet i studien, utan att behöva ange något skäl härför, utan att detta påverkar omhändertagandet i övrigt.

______________________________ ____________________

Patientens underskrift Datum

______________________________ ____________________

Anhörigs underskrift Datum

______________________________ ____________________

Studieansvarigs underskrift Datum

Vid frågor kontakta Anna Strömberg tel 013-227762, mini-call 0740-405951,
Kardiologiska kliniken, Universitetssjukhuset i Linköping
