# Supplementary material for: Long Term Follow-Up after a Randomized Integrated Educational and Psychosocial Intervention in Patient-Partner Dyads Affected by Heart Failure
Source: PLoS One. 2015 Sep 25;10(9):e0138058. doi: 10.1371/journal.pone.0138058 (PMC4583392; doi:10.1371/journal.pone.0138058)
Supplement: S2 File — (DOC) [file pone.0138058.s002.doc]

### EFFECTS OF EDUCATION AND PSYCHOSOCIAL SUPPORT TO

### HEART FAILURE PATIENT AND CAREGIVER DYAD

**SUMMARY OF THE RESEARCH FIELD**

# Vulnerability of Elderly HF Patients and Their Caregivers

HF is a health care epidemic that affects approximately 300 000 Swedes^5^ and close to 5 million Americans.^6^ The advancement of technologies in the treatment of cardiac events has resulted in a population of HF patients who are old and have complex needs. Mortality as well as morbidity is high and HF is the single most frequent cause of hospitalisation among persons over 65 years of age. Moreover, despite advances in the pharmacotherapy of HF up to 50% of the patients are readmitted within 6 months of initial hospital discharge.^7^

The magnitude of the problem of HF as well as the efforts to decrease costs has resulted in shorter hospital lengths of stays. With shortened lengths of stay, health professionals have less time to identify elderly HF patient’s needs for follow-up care and less time to arrange this care. Eighty percent of elderly discharged home following acute care hospitalisation claimed their informational needs were not met.^8^

The period following discharge for an acute episode of HF decompensation can be very stressful for elderly patients because of alterations in routines due to changes in their treatment plan. Caregivers are called upon to assume an increasingly important role in providing assistance to the patients during post-hospital recovery and have to adjust to new problems and expectations. Elderly caregivers may not have the resources to meet the complex caregiving needs of HF patients following discharge from the hospital if they do not receive adequate support and sufficient education. Caregiving tasks include participation in medical decision-making, symptom monitoring and management as well as promotion of adherence to treatment and lifestyle changes.^9^ Given this caregiving context, caring for a person with HF is likely to have a marked impact on caregivers that places them at risk for mental and physical health problems. Caregivers reported emotional distress that stemmed from fear over loss of their love one and lack of knowledge about the condition and treatment plan. They also faced changes in their lives that affected their daily routines including decreased time for relationship with others and for themselves. The extent to which caregivers perceive their emotional or physical health, social life and financial status has suffered as a result of caring for a relative has been referred to as caregiver burden. A high caregiver burden has been shown to compromise caregivers’ health and health-promoting behaviors.^10^

#### An Integrated Care Program for Patient-Caregiver Dyads

Spouses of HF patients who were given attention and were included in the plan of care reported more positive experiences as caregivers.^2^ Caregiver QOL is particularly important when they are providing care for elderly persons because often the caregiver serves as proxy for elderly who are too sick to participate in planning for discharge. It has been shown that elderly patients and their caregivers are better prepared to manage their care following hospitalisation when they participate in planning their discharge and are able to understand their plan of care. However, few resources are routinely available to help couples cope with the distress caused by an acute illness event.^11^ There is a need for interventions that will help patient-caregiver dyads cope with post-discharge needs and alleviate burden.

HF patients have less than adequate knowledge about their HF and its treatment.^12^ Likewise, caregivers consistently expressed a need for information about the elder’s condition, medications, diet, and signs of complications. Although knowledge is not sufficient to influence health behaviour, it is necessary to produce it. Teaching the patient-caregiver dyad about the disease and how it is managed and emphasizing the importance of participation in management decisions and daily care, allows the patient-caregiver dyad to “take charge” of the patient’s illness, thus fostering self-efficacy and a sense of control. Patients are also often faced with the need for support to make and maintain difficult lifestyle modifications and prevent complications and deterioration. On the other hand, caregivers need assistance caring for disabled family members. Therefore, interventions that support patients to make lifestyle modifications and help caregivers cope with providing care are necessary to accommodate limitations in both the patients’ and caregivers’ adjustment to illness and changes in the treatment plans.

The beneficial effects of HF management programs on patient outcomes are well supported in the literature. Providing patients with education about disease and treatment, emotional counselling, close monitoring and follow-up decreased mortality, morbidity, hospital costs, improved medication adherence and enhanced QOL.^13-17^ No studies have focused on interventions for caregivers who need to cope with the distress of managing care for the elderly HF patient.

To address the gaps in current research an integrated cognitive-behavioural framework that focuses on problem solving, information acquisition, self-care management for symptoms, and emotional and social support for patient-caregiver dyads is therefore proposed. Cognitive, psychosocial and behavioural strategies assist dyads in recognizing and modifying factors that contribute to physical and emotional distress by changing thoughts and behaviours in a positive manner and assisting dyads in solving problems related to implementing strategies for self-care.^18^ Computer-based education has been shown to be feasible and effective in terms of improving knowledge in elderly patients with HF^19-20^ and therefore a combined nurse-led and computer-based intervention will be tested and compared to care as usual in this study.

### SPECIFIC AIMS

Patients with chronic heart failure (HF) have debilitating symptoms associated with psychological stress and burden on both them and their caregivers.^1^ HF has several negative consequences for the family and caregivers.^2-5^ and caregiver characteristics has been shown to affect patient outcomes, for example the emotional well-being of caregivers has been shown to be an independent predictor of emotional well-being among HF patients.^2-5^ These findings inspired us to develop and test an integrated care program for HF patients and their caregivers. The overall goal of the proposed project is to test the effectiveness of an integrated care program that combines cognitive, psychosocial and behavioural therapy designed for patient-caregiver dyads and is delivered in a face-to-face and computer-based format aiming to improve patient and caregiver outcomes.

# Primary Aim

1. To determine the effectiveness of an integrated care program that combines education and psychosocial support compared to care as usual on time to first event (death or rehospitalisation), quality of life (QOL), emotional well-being, caregiver burden and perceived control in elderly patients with chronic HF and their partners.

### RESEARCH DESIGN AND METHODS

##### ***Study Design and Procedures***

The proposed study will use a randomised, controlled design. All participants, regardless of random assignments will receive information related to their HF treatment and optimised medical care as prescribed by their physician. The participants in the experimental group (Exper) will participate in an integrated care intervention, delivered in three modules through nurse-led face-to-face counselling, a computer-based CD-ROM program and other written teaching materials. (Table 1). Each module is designed to incorporate information acquisition and development of problem solving skills to assist dyads in recognizing and modifying factors that contribute to physical and emotional distress by changing thoughts and behaviours in a positive manner and assisting dyads in solving problems related to implementing strategies for self-care.

**Table 1: Content of each of the three modules utilized in the intervention**

|  | **Module 1** | **Module 2** | **Module 3** |
| --- | --- | --- | --- |
| **Cognitive**  **Component** | The circulatory system, definition of HF, medications and symptom management | Lifestyle modifications; diet, smoking cessation, alcohol, immunization, regular exercise | Directing the care,  relationship and sexual activities, prognosis |
| **Cognitive**  **Outcomes** | Increased knowledge on the HF syndrome and treatment | Increased knowledge on the rationale for lifestyle changes | Increased knowledge on HF care and outcomes |
| **Support Component** | Introduce psychosocial support concept | Assess patient’s need of support Modify caregiver behaviour | Assess caregiver’s need of support Discuss caregiver burden |
| **Support**  **Outcomes** | Improved awareness of the psychosocial support concept | Reduced criticism and overprotection in caregiver Improved support for self-care behaviour | Improved mutual support Decreased caregiver burden Improved control |
| **Behavioural Component** | Intentions, abilities and self-efficacy regarding self-care | Barriers to lifestyle modifications | Strategies to improve or maintain self-care behaviour |
| **Behavioural**  **Outcomes** | Daily weighting Monitoring of symptoms Flexible diuretic intake Adherence with medications | Salt and fluid restriction Influenza and Pneumococcal immunizations Regular Exercise | Identifying life priorities and planning for the future |
| **Teaching material** | Booklet 1 - basic level Flip chart | Booklet 2 – advanced level CD-ROM | Booklet 2– advanced level CD-ROM |

The first session will be conducted at the hospital prior to discharge. The two remaining sessions will be conducted at the dyads’ homes or in the heart failure clinic at 2 and 6 weeks following discharge. The place will be chosen by the patient-caregiver dyad. Each session will last approximately 60 minutes. Participants in Exper will receive follow up and reinforcement of the intervention after 12 weeks. The follow-up will include assessment and counselling of cognitive, support and behavioural outcomes and other related issues. The dyad will also be offered repeated computer-based education. The integrated care program will cover a 3-month period and follow up will be 24 months (Table 2).

**Table 2: Schedule of intervention and evaluation times**

| **Time** | **Baseline** | **Week 2** | **Week 6** | **Week 12** | **Week 52** | **Week 104** |
| --- | --- | --- | --- | --- | --- | --- |
| **Exper** | T1/M 1 | M 2 | M3 | F1/T2 | T3 | T4 |
| **Control** | T1 | X | X | T2 | T3 | T4 |

# Exper (face-to-face and computer-based intervention), Control (care as usual)

T1 (evaluation time 1); T2 (evaluation time 2); T3 (evaluation time 3); T4 (evaluation time 4)

M1 (module 1); M2 (module 2); M3 (module 3); F1 (follow up and reinforcement)

***Sample, Sampling Plan, and Setting***

The project will include a pilot study and a main study. Initially, we will enrol 40 patients. This sample size will not provide the power needed to test the hypotheses. However, the goal of the pilot is to test feasibility of the intervention and the battery of instruments and collect data needed to verify the preliminary sample size calculation for the main study.

Potential participants will be recruited from Sweden at Linköping University Hospital and Department of Medicine, Norrköping County Hospital, Norrköping, using a consecutive sampling procedure. Patient eligibility criteria include age ≥18 years old; able to speak and understand Swedish; admission to hospital due to worsening HF symptoms or HF decompensation and to be discharged home with a family caregiver at home. Caregiver eligibility include being a person who is in a continuous relationship with the patient, has primary responsibility for the care of the patient and is able to speak and understand Swedish. Patient-caregiver dyads will be excluded for the study if either the patient or caregiver has a life-threatening comorbidity or neurological disorder that impairs cognition.

The development of the intervention will be done during the spring 2004. Enrolment of patients and follow up in the pilot study is planned to start 2004-05-01. After data analyses and calculation of sample size, the main study will start in the beginning of 2005.

***Instruments***

Data will be collected with the following instruments at baseline, after 3, 12 and 24 months. All instruments are available in a validated English and Swedish version.

Sociodemographic Form: A questionnaire will be used to identify gender, ethnicity, education, income, marital status, living status, and type of health insurance status.

Health History: A medical record review form will be completed at the time of enrolment for both patients and caregivers. For the patient it will include data on cardiac history (e.g. length of time since they had been diagnosed with HF), co-morbidities, cardiac risk factors, medications, and length of hospital stay. For the caregiver it will include data on presence of cardiac risk factors, cardiac or other chronic disease and medications.

Clinical event History: Information related to death, unscheduled physician visits, emergency room visits and hospitalisations will be registered during time of follow up.

Short Form-36: A generic 36-item scale which evaluates 8 health concepts: physical functioning, bodily pain, role limitations due to physical health problems, role limitations due to personal or emotional problems, emotional well-being, social functioning, energy/fatigue, general health perceptions, as well as a single item for overall appraisal of change in perceived health within the last year. The time frame for appraisal of all of the items is within the past 4 weeks.^21^

Perceived Control: A 4-item questionnaire designed to assess perceived control in cardiac patients. Responses are scored on a Likert scale from 1 (none) to 5 (very much). Total score ranges from 4 to 20, with higher scores reflecting increased feelings of perceived control. Cronbach´s α for the scale was 0.89.^22^

Caregiver Burden Scale: A 22-item scale evaluating different types of caregiver burden. Responses are scored on a Likert scale from 1 (none) to 5 (very much). Total score ranges from 0 to 66, with higher scores reflecting increased caregiver burden.^2^3

Self-Care Behaviour Scale: A 12-item scale that utilizes a 5-point Likert scale between 1 (I completely agree) and 5 (I completely disagree) to measure self-care behaviours related to HF. Overall score ranges between 12-60; lower scores indicate better self-care behaviour. Cronbach´s α for the scale was 0.71.24-^2^5

Data Analysis

Statistical analysis will be performed using SPSS release 11.0 for Windows. To examine the equality of the 2 groups, baseline sociodemographic and clinical characteristics will be compared using chi-square or t-tests depending on the level of measurement. Randomisation should ensure equal distribution between the 2 groups of potentially confounding characteristics. However, if there are significant differences between the groups on crucial characteristics, these will be used as factors or covariates as appropriate in subsequent analyses.

The effect of the intervention on each of the variables of interest will be evaluated at the aggregate level by a t-test to determine if the control and experimental group have significantly different mean scores on all variables of interest at baseline, 3, 12 and 24-months. The mean changed scores for the control and experimental group will also be compared using a t-test. Between groups differences in the variables of interest from baseline to 3 and 12-months will be measured using a repeated measures ANOVA to characterize differences between groups at each time period and differences within groups across time. The relationship between sociodemographic and clinical characteristics and each of the variables of interest will be analysed using Pearson moment correlations for normally distributed scores or Spearman rank order correlation for non-normally distributed scores. Multiple regressions will be used to determine the relationship among independent variables and QOL, emotional well-being, and perceived control.

Health economical analysis will be performed in order to evaluate the cost-effectiveness of an integrated care program that combines cognitive, psychosocial, and behavioural therapy delivered in a face-to-face and computer-based format and the care as usual. The costs of the interventions as well as health care utilization will be calculated.

Ethical Considerations

Permissions for the study has been approved by the Regional Ethics Committees for Human Research at the Linköping University (Dnr 03-569) and from the UCLA Office for Protection of Research Subjects. All subjects will receive both verbal and written information about the study and be asked to sign an informed consent prior to inclusion.

A potential risk of the study is that some patients and caregivers may experience anxiety after the delivery of the intervention. Patients and caregivers will fill in questionnaires at baseline, after 3, 12 and 24 months. Some questions may make participants feel uncomfortable. The length of the paper work and intervention (approximately 60/minutes/session) may be fatiguing for some participants. As with any research study, there are potential risks related to subjects’ privacy and confidentiality. In an effort to minimize risks, every step will be taken to make participants feel comfortable when completing study procedures. Patients and caregivers will be told that they may refrain from answering questions and may refuse the procedures as well as withdraw from the study at any time.

All participants, regardless of random assignments will receive information related to HF and treatment. The experimental group will receive additional assistance, above and beyond what is offered by the formal healthcare system. The anticipated benefits to patients and caregivers in the experimental group are potentially substantial, since they will receive information and coaching that may improve their outcomes. While no direct benefit to patients in the control group is anticipated, the knowledge obtained from the study may provide critical evidence about the importance of educating caregivers and elderly patients with HF. Since the interventions are not standard care, the ethical demands of doing good, not harming and being fair are taken into consideration.

**SIGNIFICANCE OF THE STUDY**

Patients with HF and their caregivers will continue to be a large group in the health care system. It is therefore of great importance both from the perspective of the individuals and due to increasing health care costs to design and evaluate new interventions in order to reduce the need for hospitalisation and improve QOL and well-being in patients and caregivers. This study will evaluate if an integrated care program that combines cognitive, psychosocial and behavioural therapy designed for patient-caregiver dyads can improve patient and caregiver outcomes and decrease health care costs. If this type of intervention is successful it should be considered after hospitalisation due to HF and perhaps also implemented in primary health care. The possibilities of using a computer-based intervention will also be valuable to evaluate since patients and their caregivers can be educated with a less involvement from health care professionals.

***Project group***

**Anna Strömberg**, assistant professor, senior lecturer, IMV, Hälsouniversitetet, Linköping, clinical nurse specialist, Department of Cardiology, Linköping University Hospital.

**Ulf Dahlström,** professor, IMV, Hälsouniversitetet, Linköping, chief physician, Department of Cardiology, Linköping University Hospital.

**Susanna Ågren,** PhD student, IMV, Hälsouniversitetet, Linköping, intensive care nurse, Department of Cardiology, Linköping University Hospital.

**CHALLENGE AND PUBLICATION OF RESULTS**

None of the researchers in the study has bonds or challengeability that can affect design or publication of results. Results from the study will be published in scientific journals and will generate material for at least one PhD thesis.

###### REFERENCES

1. Karmilovich S. Burden and Stress Associated with Spousal Caregiving for Individuals with Heart Failure. *Progress in Cardiovascular Nursing* 1994;**9**:33-8.

2. Martensson J, Dracup K, Fridlund B. Decisive situations influencing spouses' support of patients with heart failure: a critical incident technique analysis. *Heart Lung* 2001;**30**:341-50.

1. Evangelista L, Dracup K, Doering L, Westlake C, Hamilton MA, Fonarow G. Emotional well-being of heart failure patients and their caregivers. *J Card Fail* 2002;**8**:300-5.
2. Martensson J, Dracup K, Canary C, Fridlund B. Living with heart failure: depression and quality of life in patients and spouses*. J Heart Lung Transplant.* 2003 Apr;**22**:460-7.

5. Ryden-Bergsten T, Andersson F. The health care costs of heart failure in Sweden. *J Intern Med* 1999;**246**:275-284.

6. American Heart Association. 2002 Heart and Stroke Statistical Update. Dallas, Texas, American Heart Association.

7. Krumholz HM, Parent EM, Tu N, Vaccarino V, Wang Y, Radford MJ *et al*. Readmission after hospitalization for congestive heart failure among medicare beneficiaries. *Archives of Internal Medicine* 1997;**157**:99-104.

8. Mistiaen P, Duijnhouwer E, Wijkel D, DeBont M, Vegeer A. The problems of elderly people at home 1 week after discharge from the acute setting. *J Adv Nurs* 1997;**25**:1233-40.

9. Dracup K, Baker D, Dunbar S, Dacey RA, Brooks NH, Johnson JC *et al*. Management of heart failure: counseling, education and lifestyle modifications. *JAMA* 1994;**272**:1,442-1,446.

10. Faison KJ, Faria SH, Frank D. Caregivers of chronically ill elderly: Perceived burden. *J Comm Health Nurs* 1999;**16**:243-53.

11. Daugherty J, Saarmann L, Riegel B, Sornborger K, Moser D. Can we talk? Developing a social support nursing intervention for couples. *Clin Nurse Spec* 2002;**16**:211-8.

12. Ni H, Nauman D, Burgess D, Wise K, Crispell K, Hershberger RE. Factors influencing knowledge of and adherence to self-care among patients with heart failure. *Archives of Internal Medicine* 1999;**150**:1613-9.

13. Stewart S, Vandenbroek AJ, Pearson S, Horowitz JD. Prolonged beneficial effects of a home-based intervention on unplanned readmissions and mortality among patients with congestive heart failure. *Arch Intern Med* 1999;**159**:257-61.

1. Naylor MD, Brooten D, Campbell R, Jacobsen BS, Mezey MD, Pauly MV *et al*. Comprehensive discharge planning and home follow-up of hospitalized elders. *JAMA* 1999;**281**:613-20.

15. Strömberg A, Mårtensson J, Fridlund B, Levin L-Å, Karlsson J-E, Dahlström U. Nurse- led heart failure clinics improve survival and self-care behaviour in patients with heart failure. Results from a prospective, randomised study. *European Heart Journal* 2003;24(11):1014-23.

16. McAlister FA, Lawson FM, Teo KK, Armstrong P. A systematic review of randomized trials of disease management programs in heart failure. *Am J Med* 2001;**110**:378-84.

17. Blue L, Strong E, McMurray J, Davie A, McDonagh TA, Murdoch DR *et al*. Randomised controlled trial of specialist nurse intervention in heart failure. *BMJ* 2001;**323**:715-8.

18. Bandura A. Self-efficacy: Toward a unifying theory of behavioral change. *Psychol Rev* 1977;**84**:191-215.

1. Strömberg A, Dahlström U, Fridlund B. Computer-based education for patients with chronic heart failure. A randomised, controlled, multicentre study of the effects on knowledge, compliance and quality of life. *Journal of Advanced Nursing* **in press**
2. Strömberg A, Ahlén H, Fridlund B, Dahlström U. Interactive education on CD-ROM - a new tool in the education of heart failure patients. *Patient Education and Counselling* 2002; **46**:75-81.
3. Ware JE. SF-36 health survey manual and interpretation guide. Boston: The New Health Institute, New England Medical Center, 1993.
4. Moser DK, Dracup K. Psychosocial recovery from a cardiac event: the influence of perceived control. Heart lung 1995;24:273-280-
5. Elmståhl S, Malmberg B, Annerstedt L. Caregiver’s burden of patients 3 years after stroke assessed by a novel caregiver burden scale. Arch Phys Rehabil 1996;77:177-182.
6. Jaarsma T, Halfens R, Abu-Saad HH, Dracup K, Stappers J, van Ree J. Effect of education and support on self-care and resource utilization. Eur Heart J 1999;20:673-682.
7. Jaarsma T, Strömberg A, Mårtensson J, Dracup K. The European Heart Failure Self-Care Behaviour Scale: ready to use. *European Journal of Heart Failure* 2003;5(3):363-70
8. Strömberg A, Mårtensson J. Gender differences in patients with heart failure. *European Journal of Cardiovascular Nursing* 2003;2:7-18.
